# CmHRE2L-CmACS6 transcriptional cascade negatively regulates waterlogging tolerance in Chrysanthemum

**DOI:** 10.1186/s43897-024-00138-8

**Published:** 2025-03-03

**Authors:** Yajun Yan, Wanwan Zhang, You Wang, Yue Wang, Chuanwei Li, Nan Zhao, Lijie Zhou, Jiangshuo Su, Likai Wang, Jiafu Jiang, Sumei Chen, Fadi Chen

**Affiliations:** State Key Laboratory of Crop Genetics & Germplasm Enhancement and Utilization, Key Laboratory of Landscaping, Ministry of Agriculture and Rural Affairs, Key Laboratory of Biology of Ornamental Plants in East China, National Forestry and Grassland Administration, College of Horticulture, Nanjing Agricultural University, No.1 Weigang, Nanjing, 210095 China

**Keywords:** Waterlogging stress, Chrysanthemum, ACS, ERF, Ethylene biosynthesis

## Abstract

The role of ethylene as an initial signaling molecule in waterlogging stress is well-established. However, the complex molecular mechanisms underlying ethylene biosynthesis and its functional significance in chrysanthemums under waterlogging conditions have remained unclear. In this study, we observed an increase in the expression of *1-aminocyclopropane-1-carboxylate synthase 6* (*CmACS6*), which encodes a key enzyme responsible for ethylene biosynthesis, in response to waterlogging. This elevation increases ethylene production, induces leaf chlorosis, and enhances the chrysanthemum's sensitivity to waterlogging stress. Moreover, our analysis of upstream regulators revealed that the expression of *CmACS6*, in response to waterlogging, is directly upregulated by CmHRE2-like (Hypoxia Responsive ERF-like, CmHRE2L), an ethylene response factor. Notably, CmHRE2-L binds directly to the GCC-like motif in the promoter region of *CmACS6*. Genetic validation assays demonstrated that *CmHRE2L* was induced by waterlogging and contributed to ethylene production, consequently reducing waterlogging tolerance in a partially *CmACS6*-dependent manner. This study identified the regulatory module involving CmHRE2L and *CmACS6*, which governs ethylene biosynthesis in response to waterlogging stress.

**Supplementary Information:**

The online version contains supplementary material available at 10.1186/s43897-024-00138-8.

## Core

CmHRE2L binds to the GCC-like motif in the promoter region of *CmACS6*, directly enhancing* CmACS6* expression under waterlogging conditions in chrysanthemum ‘Jinba’. The increased expression of *CmACS6* led to an ethylene surge near the leaves above water, subsequently inducing leaf chlorosis. Genetic validation assays confirmed that the CmHRE2L-CmACS6 transcriptional cascade negatively influences chrysanthemum ‘Jinba’ waterlogging tolerance.

## Gene & Accession Numbers

CmACS6 accession: cmak_058780 (chrysanthemum genome database-http://210.22.121.250:8880/asteraceae/homePage).

CmHRE2-like accession: BankIt2738594. (need to add reference-Wang, 2024, Bmc Plant Biol)

## Introduction

Waterlogging represents a significant natural disaster affecting numerous countries globally and is a prevalent agro-meteorological challenge in China (Yin et al. 2023). During waterlogging stress, soil oxygen content rapidly diminishes to approximately 1/10000 of normal growing conditions, resulting in hypoxic or anoxic soil conditions. Consequently, the submerged root systems of affected plants are unable to maintain regular air contact (Sairam et al. 2009; Steffens and Rasmussen 2016), severely impacting plant growth. Thus, investigating the mechanisms of plant resistance to waterlogging stress is crucial for promoting agricultural sustainability. Ethylene, an early signal detected during waterlogging stress, is produced and accumulates in plant cells under these conditions (Hartman et al. 2019; Zandalinas et al. 2021). Ethylene signaling influences gibberellic acid, gibberellin, and auxin pathways, promoting the development of waterlogging-adapted features such as aerated tissues and adventitious roots to mitigate hypoxia (Dawood et al. 2016; Sasidharan and Voesenek 2015; Yang et al. 2015). Increased ethylene production can be observed within hours of waterlogging stress onset (Voesenek and Sasidharan 2013). It is hypothesized that elevated ethylene levels, mediated through signal transduction pathways and downstream regulation of ethylene-related genes, may induce adaptive changes at the morphological and physiological levels, enhancing plant tolerance to waterlogging conditions.

Chrysanthemum (*Chrysanthemum morifolium*) is indigenous to China and possesses significant ornamental, medicinal, and economic value. However, its shallow root system renders the chrysanthemum vulnerable to waterlogging stress. In China, the middle and lower reaches of the Yangtze River and southern regions are susceptible to short- or long-term flooding due to frequent summer rainfall (Ahsan et al. 2007; Kuai et al. 2015). Short-term flooding can severely impede the normal growth of cultivated chrysanthemums and potentially cause widespread mortality, affecting quality and economic value (Su et al. 2016). A previous study demonstrated that ethylene production in *Chrysanthemum zawadskii* (a waterlogging-tolerant wild species) was higher than in *Chrysanthemum nankingense* (a waterlogging-sensitive wild species) during waterlogging stress (Yin 2011). Yin (2011) reported that *C. zawadskii* generates more ethylene in response to waterlogging stress, and ethylene may promote programmed cell death in the root system and the formation of aerated tissues to enhance plant aeration under waterlogging stress. Ethylene production was increased by waterlogging in both chrysanthemum cultivars, '53–4' (waterlogging tolerant cultivar) and '13–13' (waterlogging sensitive cultivar). However, '53–4' exhibited an earlier and significantly higher ethylene production peak than '13–13'. It was hypothesized that the increase in ethylene in the waterlogging-tolerant cultivar may have facilitated adventitious root growth. This increased the area of oxygen uptake to liberate the plant from the low-oxygen environment during waterlogging (Yin et al. 2009). The ethylene production of 'Nannong Xuefeng' (waterlogging tolerant cultivar) plants was significantly higher than that of the waterlogging sensitive cultivar 'Qinglu' (Zhao et al. 2018). The intense ethylene synthesis response may be a crucial adaptation of the waterlogging-tolerant cultivar 'Nannong Xuefeng' in response to waterlogging and reoxygenation. These findings suggest that ethylene may play a pivotal role in the chrysanthemum waterlogging response. However, the molecular regulatory network of ethylene biosynthesis in chrysanthemum in response to waterlogging stress remains unclear. In this study, we found that *CmACS6*, a crucial gene involved in ethylene biosynthesis, exhibited a significant increase in expression under waterlogging stress. Further studies indicate that *CmACS6* plays a role in ethylene production, compromises the health of chrysanthemum leaves and increases the sensitivity of the plants to waterlogging stress. Subsequently, we found that CmHRE2-like directly targets *CmACS6*, and genetic validations showed that the role of CmHRE2-like in altering ethylene biosynthesis and waterlogging sensitivity is dependent on *CmACS6*. Therefore, the present study provides a novel insight into the response to waterlogging stress.

## Results

### Waterlogging stress elevates ethylene production in chrysanthemum

**Fig. 1:**
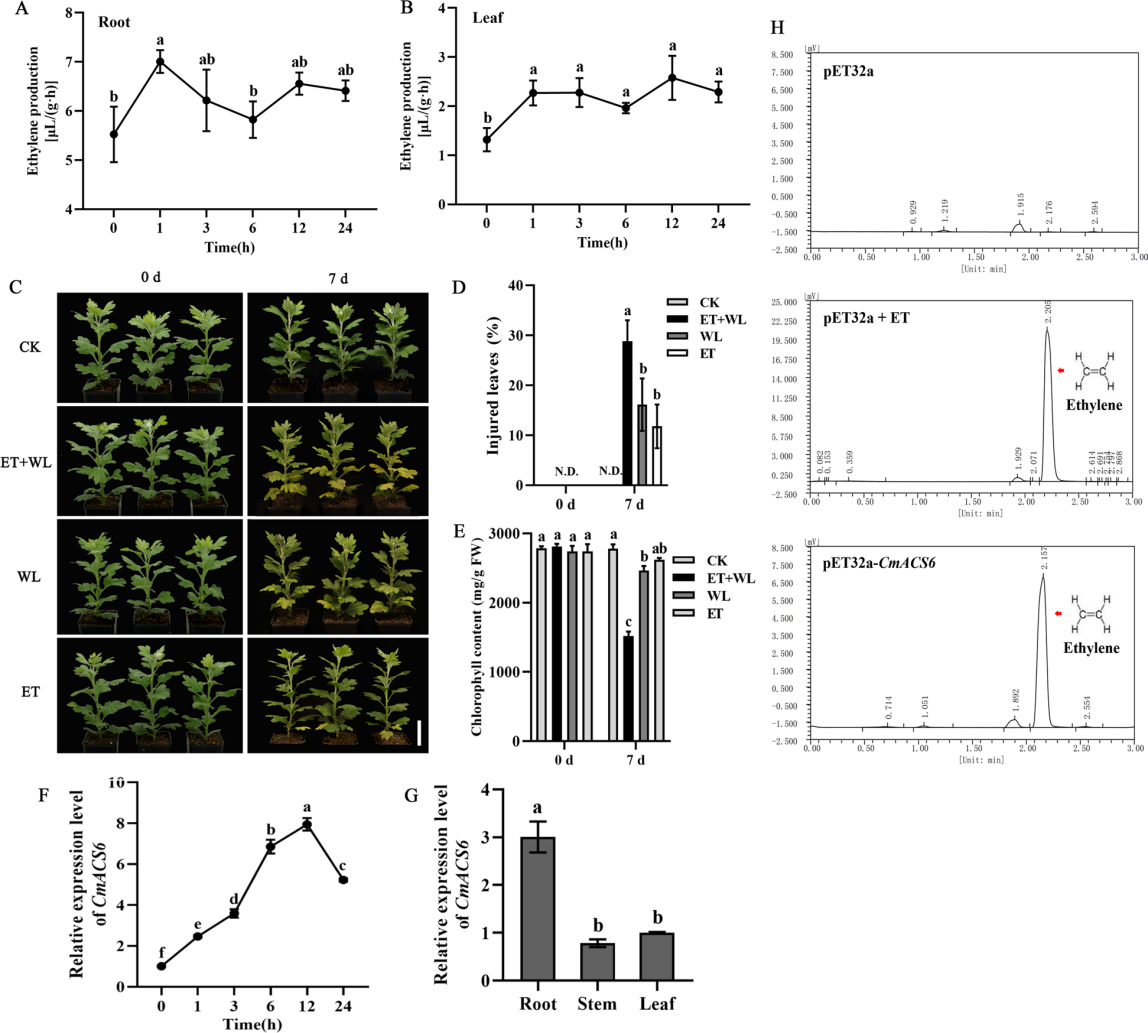
Ethylene and *CmACS6* involved in waterlogging stress response in chrysanthemum. **A-B**, Changes in ethylene production in ‘Jinba’ under waterlogging stress at various time points. **A** Ethylene production in the root. **B** Ethylene production in the root. **C** Phenotype of chrysanthemum after waterlogging treatment with/without exogenous ethylene. CK: control; ET + WL: exogenous ethylene + waterlogging treatment, WL: waterlogging treatment alone, ET: exogenous ethylene treatment alone. Bar: 5 cm. **D** Statistical analysis of the percentage of injured leaves after waterlogging stress. N.D.: Not detect. **E** Statistical analysis of chlorophyll content after waterlogging stress. **F** Expression pattern of *CmACS6* in response to waterlogging. Error bars represent standard deviation (SD, *n* = 3). Lowercase letters indicate significant differences at *P* < 0.05 (ANOVA, Turkey’s correction). **G** Expression pattern of *CmACS6* in different tissues. **H** In vitro catalytic test results of CmACS6

To investigate the changes in ethylene production in chrysanthemum 'Jinba' following waterlogging stress, we subjected the plants to waterlogging conditions. We monitored the alterations in ethylene production in both roots and leaves at various treatment intervals. The results revealed that ethylene production in root samples increased after 1 h of waterlogging treatment, reaching 1.28 times than that of the control. However, the ethylene production at other time points showed little increase and generally showed up and down fluctuations (Fig. 1A). In leaf samples, ethylene production was significantly higher (1.77 times) than the control group at all time points following waterlogging treatment, with the highest production observed after 12 h, reaching 2.01 times that of the control (Fig. 1B). Additionally, we observed that under natural conditions (untreated groups), ethylene production in roots was considerably higher (4.23 times) than in leaves (Fig. 1A, B).

Given the observed changes in ethylene production following chrysanthemum waterlogging, we sought to investigate the potential influence of ethylene on the chrysanthemum phenotype under waterlogging stress conditions. To this end, we incorporated exogenous ethylene into the chrysanthemum waterlogging stress treatment. Cut chrysanthemum 'Jinba' is a significant commercial cultivar, and the health of its above-ground components, particularly the leaves, substantially impacts its quality and value during plant production. Consequently, our investigation primarily focused on the response of chrysanthemum 'Jinba' leaves to waterlogging stress. After a 7-day treatment period, chrysanthemum plants in the 'ET + waterlogging conditions (WL)' group exhibited a severe waterlogging-sensitive phenotype. The middle and lower leaves of chrysanthemums in this group displayed visible and extensive yellowing (Fig. 1C), demonstrating a significantly higher percentage (28.78%) of injured leaves in the plants (Fig. 1D) and a reduction in chlorophyll content (Fig. 1E). While chrysanthemums in the 'WL' group also exhibited a water-sensitive phenotype, the injury was less pronounced compared to the 'ET + WL' group. These findings indicate that exogenously applied ethylene significantly enhanced the sensitivity of chrysanthemum 'Jinba' to waterlogging stress, resulting in severe leaf chlorosis.

### CmACS6 contributes to enhanced ethylene production in response to waterlogging stress

Given ethylene's crucial regulatory role in chrysanthemum's waterlogging stress response, we examined ethylene synthesis-related genes among the differential expression genes (DEGs) in the RNA-seq data of the waterlogging-tolerant cultivar 'Nannong Xuefeng (XF)' and the highly waterlogging-sensitive cultivar 'Qinglu (QL)' from a previous study (Zhao et al. 2018). The *CmACS6* gene, encoding a vital ACC synthase in the ethylene biosynthetic pathway, was significantly upregulated by waterlogging and differentially expressed in both cultivars (Figure S1). Subsequently, we evaluated the expression level of *CmACS6* gene in 'Jinba' roots following waterlogging stress. Results demonstrated that *CmACS6* responded rapidly to waterlogging (Fig. 1F), with significant upregulation detected after 1 h of stress. Under prolonged waterlogging, *CmACS6* expression peaked (7.93-fold increase) at 12 h post-stress initiation. This observation indicated that waterlogging stress significantly induced *CmACS6* gene expression. To elucidate the function of *CmACS6*, we cloned it from chrysanthemum, revealing a 1,452 bp open reading frame (ORF) encoding a 483 amino acid polypeptide (Figure S2). The gene exhibited highest expression in roots and lowest in stems (Fig. 1G).

Further investigation was conducted to determine whether the CmACS6 protein possesses ACC synthase catalytic function. The results demonstrated that the *CmACS6* gene encodes an active ACC synthase, capable of catalyzing ACC formation, thereby playing a crucial role in ethylene biosynthesis (Fig. 1H).

### *CmACS6* plays a negative role in regulating waterlogging tolerance in chrysanthemum

**Fig. 2:**
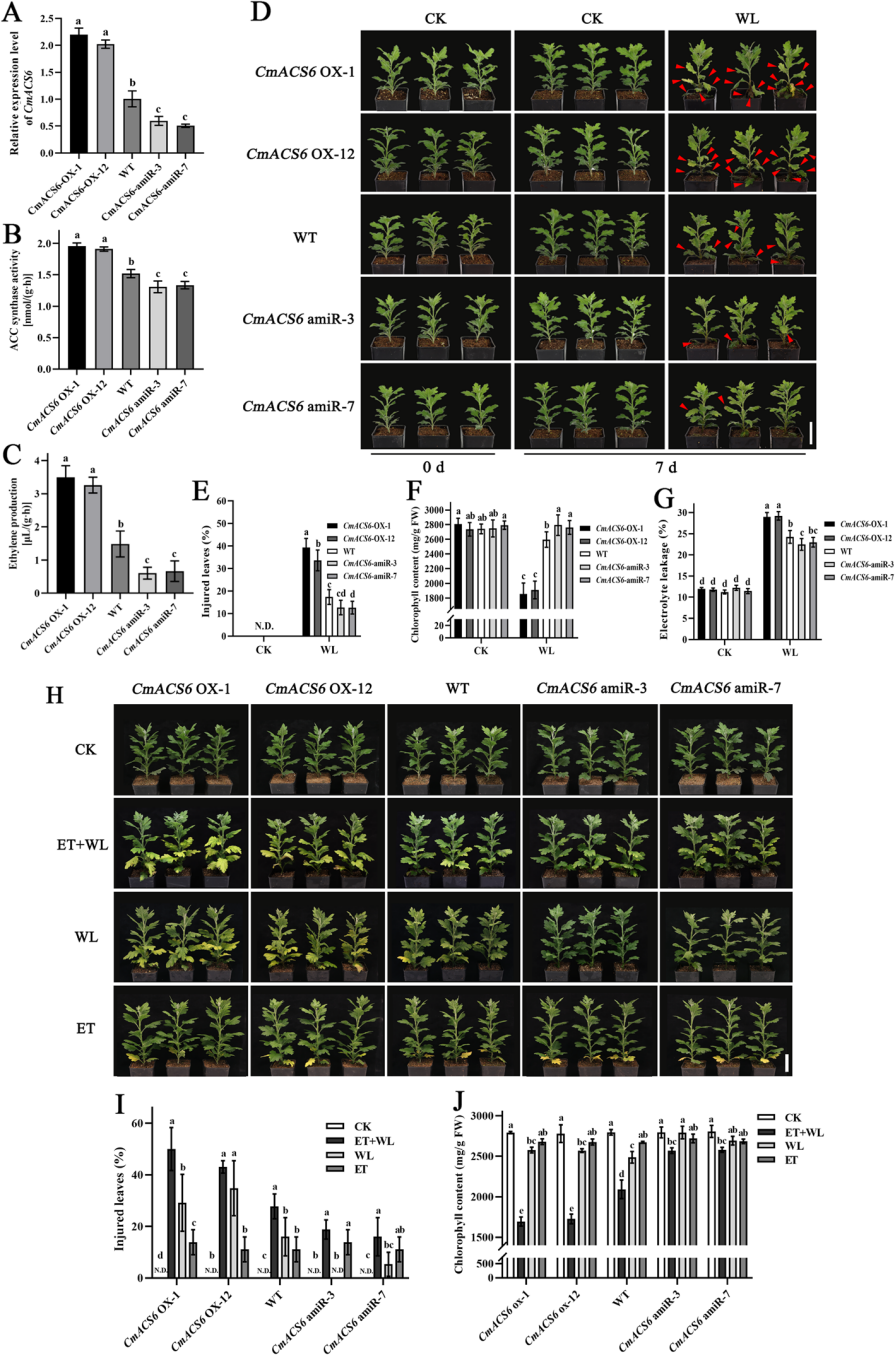
Phenotypes of *CmACS6* transgenic lines under waterlogging stress treatment (with/without exogenous ethylene application). **A** The expression level of *CmACS6* in wild-type and transgenic chrysanthemum lines. **B** ACC synthase activity of *CmACS6* transgenic chrysanthemum lines. **C** Ethylene production of *CmACS6* transgenic chrysanthemum lines. **D** Phenotype of *CmACS6* transgenic plants under natural growth conditions (0 d) and waterlogging conditions (7 d). Red arrows indicate the partially injured leaves after waterlogging stress. Bar: 5 cm. **E–G** Physiological index determinations of *CmACS6* transgenic and wild-type chrysanthemum under natural growth CK and WL. **E** Percentage of injured leaves. N.D.: Not detect. **F** Chlorophyll content. **G** Electrolyte leakage. **H** Phenotype of *CmACS6* transgenic lines after different treatments. CK: control; ET + WL: exogenous ethylene + waterlogging treatment, WL: waterlogging treatment alone, ET: exogenous ethylene treatment alone. Bar: 5 cm. **I** Percentage of injured leaves of *CmACS6* transgenic lines after treatment. N.D.: Not detect. **J** Chlorophyll content of *CmACS6* transgenic lines after treatment. Error bars represent standard deviation (SD, *n* = 3). Different lowercase letters indicate significant differences at *P* < 0.05 (ANOVA, Tukey’s correction)

We have demonstrated that CmACS6 possesses catalase activity in vitro, catalyzing the formation of ACC, which can subsequently be oxidized to ethylene. To examine whether alterations in the expression level of the *CmACS6* gene affect ACC levels in chrysanthemum 'Jinba', we developed *CmACS6* overexpression and silencing transgenic lines (Fig. 2A, Figure S3) and assessed ACC synthase activity. Relative to wild-type plants (WT), the ACC synthase activity of the *CmACS6* overexpression (*CmACS6*-OX) lines exhibited a significant increase. Conversely, the ACC synthase activity in the *CmACS6* silencing (*CmACS6*-amiR) lines showed a significant reduction (Fig. 2B). These findings indicate that CmACS6 exhibits ACC synthase catalytic activity in chrysanthemum 'Jinba', with ACC synthase activity increasing and decreasing in chrysanthemum plants following overexpression and silencing of the *CmACS6* gene, respectively.

Leaves from 'Jinba' chrysanthemum *CmACS6* transgenic lines and wild-type (WT) plants were analyzed for endogenous ethylene production, with results presented in Fig. 2C. The analysis revealed that, in comparison to WT plants, ethylene production was significantly elevated in the *CmACS6*-OX lines and diminished in the *CmACS6*-amiR lines.

To investigate the role of the *CmACS6* gene under waterlogging stress in chrysanthemum, we subjected *CmACS6* transgenic and wild-type plants to waterlogging stress. After 7 days of waterlogging, the *CmACS6*-OX lines demonstrated a pronounced waterlogging-sensitive phenotype, characterized by more severe yellowing, rot, and wilting of the middle and lower leaves compared to wild-type plants (Fig. 2D). The *CmACS6*-OX-1 and *CmACS6*-OX-12 exhibited a higher percentage of injured leaves (39.35% and 33.63%, respectively) than the wild type (17.35%) (Fig. 2E). The *CmACS6*-OX lines also displayed reduced chlorophyll content and increased electrolyte leakage (Fig. 2F, G), indicating substantial leaf cell damage. In contrast, *CmACS6*-amiR lines showed improved plant phenotype, lower injury rates, higher chlorophyll content, and reduced leakage, suggesting diminished damage after *CmACS6* silencing (Fig. 2D-G). Phenotypes of transgenic lines in the field after exposure to heavy summer rains were consistent with the laboratory results (Figure S4). Additionally, the *CmACS6*-OX lines exhibited a significant decline in root volume, root length, and root tip number following exposure to waterlogging stress. Conversely, the root system of the *CmACS6-*amiR lines maintained a relatively healthy condition after waterlogging stress (Figure S5). Considering previous reports (Eun et al. 2019) indicating that overexpression of *AtACS11* reduces root length in *Arabidopsis*, it is plausible that the root system of the *CmACS6*-OX lines may be less developed than that of the wild type, potentially contributing to the tolerance of the transgenic plants to waterlogging. These findings suggest that the *CmACS6* gene negatively regulates waterlogging tolerance in chrysanthemums.

### Exogenous ethylene plays a negative role in regulating waterlogging tolerance in chrysanthemum

Given that *CmACS6*-OX lines produced significantly more ethylene than the wild type and exhibited a more sensitive phenotype under waterlogging stress, we sought to verify whether exogenous ethylene under chrysanthemum waterlogging stress elicits the same response as endogenous ethylene produced by *CmACS6*-OX lines. We supplemented exogenous ethylene to the waterlogging treatment of *CmACS6* transgenic lines and subsequently observed the phenotypic changes. The resulting phenotypes are depicted in Fig. 2H. Under 'ET + WL' treatment, the *CmACS6*-OX lines demonstrated high waterlogging sensitivity, with 50.00% and 43.06% injured leaves in OX-1 and OX-12 respectively, and reduced chlorophyll content (Fig. 2I-J). In contrast, the *CmACS6*-amiR lines exhibited a more tolerant phenotype across all treatment groups, with a significantly lower percentage of injured leaves (18.80% and 16.03%) and chlorophyll loss (Fig. 2I, J). These results indicate that exogenously applied ethylene induced severe leaf chlorosis in chrysanthemum 'Jinba' and could induce a waterlogging-sensitive phenotype in *CmACS6*-amiR lines under waterlogging stress, suggesting that exogenous ethylene could compensate for the reduced ethylene production after *CmACS6* silencing. This compensation led to the accumulation of ethylene in the plant, resulting in yellowing and senescence of chrysanthemum leaves. In conclusion, exogenous ethylene application exacerbated the sensitive phenotype of chrysanthemum 'Jinba' under waterlogging stress, further supporting the notion that ethylene negatively regulates waterlogging tolerance in chrysanthemum 'Jinba'.

### Transcriptome sequencing revealed differences in waterlogging-related pathways in *CmACS6* transgenic lines of chrysanthemums

**Fig. 3:**
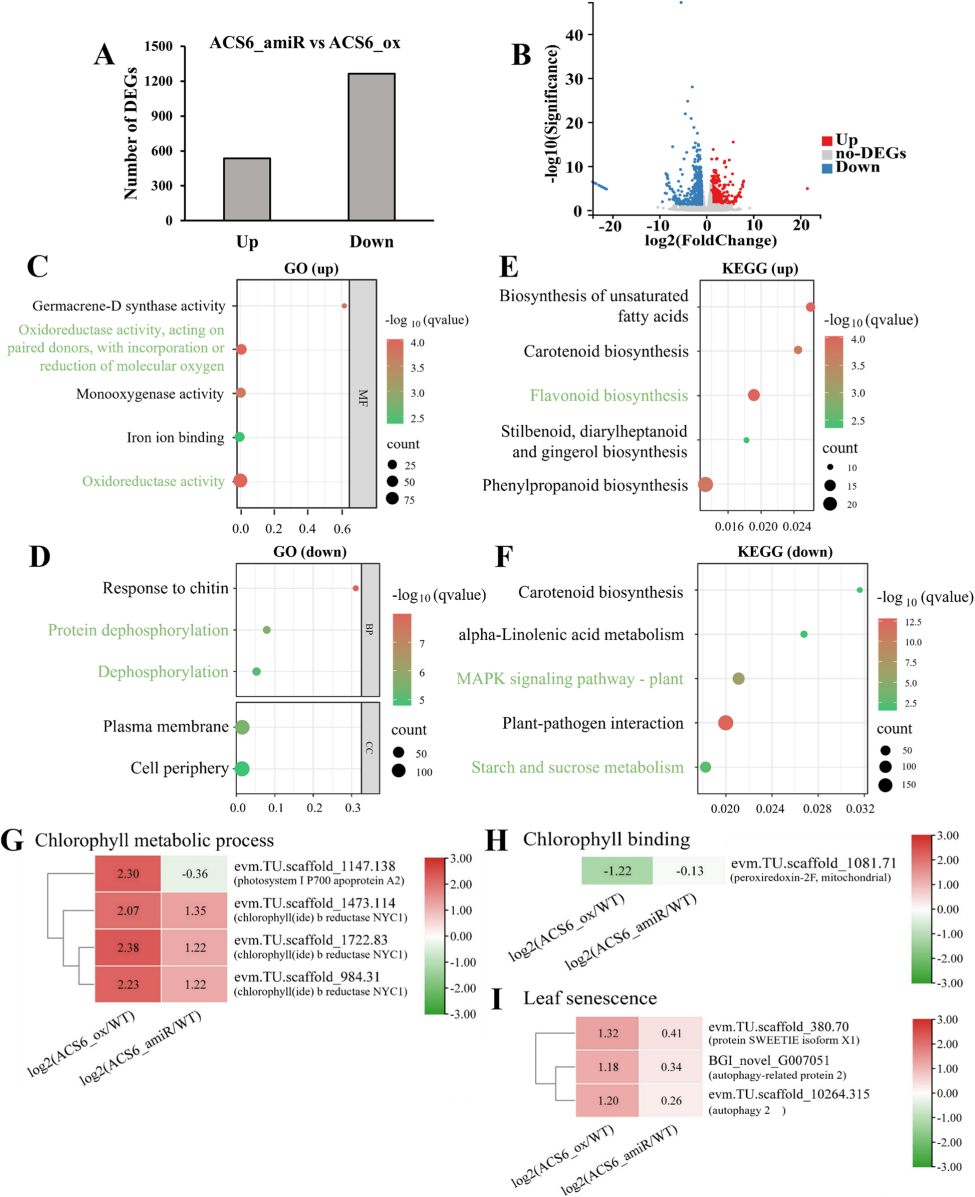
RNA-seq analysis of *CmACS6* transgenic lines (*CmACS6* amiR vs *CmACS6* OX). **A** Quantity of DEGs between *CmACS6* transgenic lines. **B** Volcano plot illustrating DEGs. **C-D** Gene Ontology (GO) enrichment analysis for upregulated (**C**) and downregulated (**D**) DEGs. **E–F** KEGG pathway enrichment analysis for upregulated (**E**) and downregulated (**F**) DEGs. The X-axis denotes the enrichment ratio. **G-I** Heatmaps depicting DEGs associated with chlorophyll metabolic processes and leaf senescence in *CmACS6* transgenic lines. **G** DEGs related to chlorophyll metabolic processes. **H** DEGs related to chlorophyll-binding proteins. **I** DEGs associated with leaf senescence

Transcriptome sequencing of the *CmACS6* transgenic chrysanthemum lines revealed that, compared with *CmACS6*-OX lines, the *CmACS6*-amiR lines exhibited 1,801 DEGs, comprising 536 up-regulated and 1,265 down-regulated genes (Fig. 3A, B). The up-regulated DEGs were predominantly enriched in oxidoreductase activity (GO enrichment) and flavonoid biosynthesis pathway (KEGG enrichment), which serve to scavenge reactive oxygen species produced in *CmACS6*-amiR lines due to waterlogging stress, thereby maintaining a balanced ROS level (Fig. 3C, D). Conversely, the down-regulated DEGs were enriched in protein dephosphorylation (GO enrichment) and MAPK signaling pathway (KEGG enrichment), indicating that phosphorylation and dephosphorylation processes might play crucial roles in *CmACS6*-amiR lines' response to waterlogging stress. Additionally, DEGs were enriched in starch and sucrose metabolism pathways, suggesting that *CmACS6*-amiR lines maintain low energy metabolism under waterlogging conditions to conserve starch and sucrose (Fig. 3E, F). In comparison to wild-type 'Jinba', chlorophyll metabolic process genes and leaf senescence genes were significantly up-regulated in *CmACS6*-OX lines, while the chlorophyll-binding gene was down-regulated in these lines (Fig. 3G-I). All clean data can be downloaded from NCBI (BioProjects: PRJNA1197569).

### *CmACS6* is directly regulated by CmHRE2-like

**Fig. 4:**
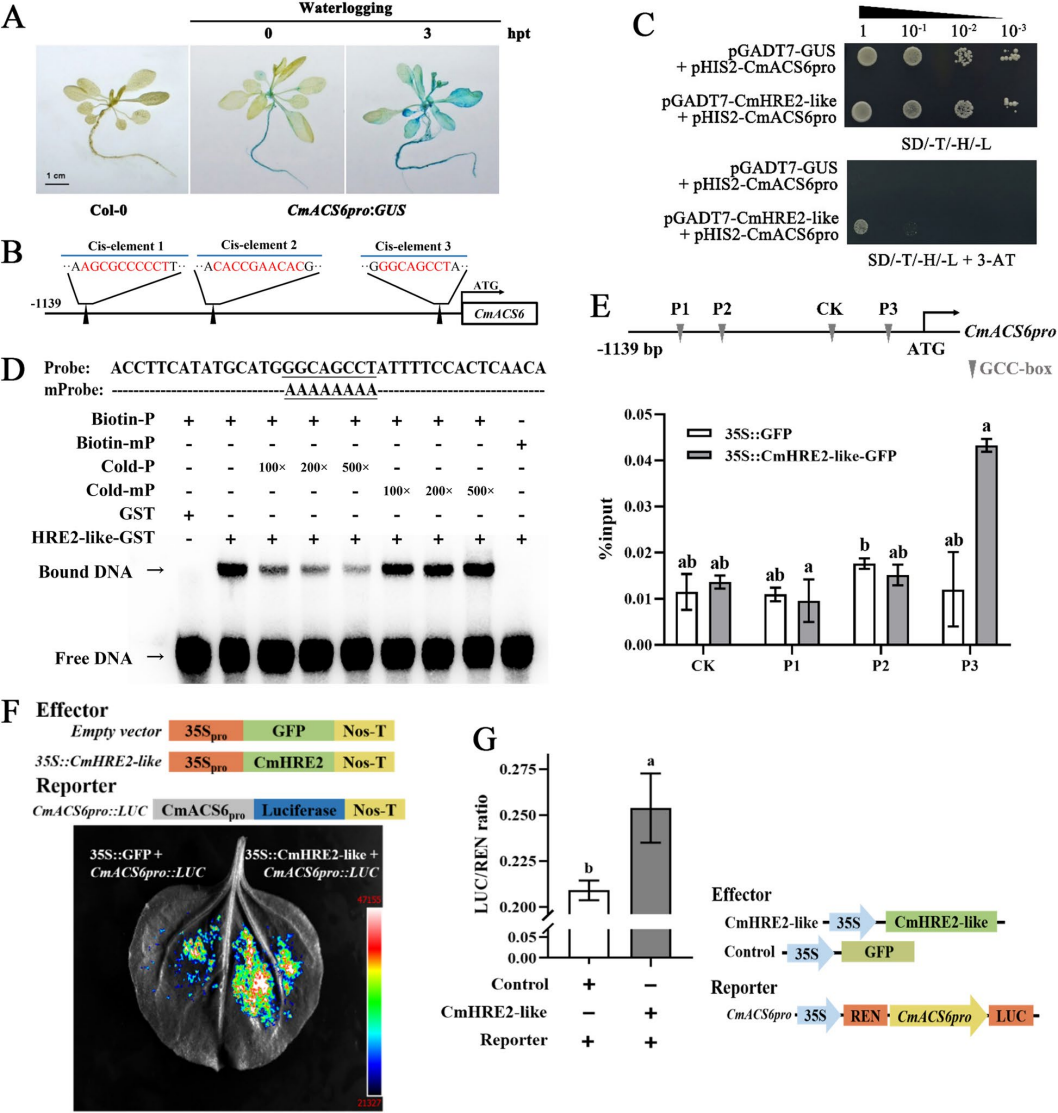
CmHRE2-like directly activates *CmACS6* genes in chrysanthemum. **A** Heterologous expression of *CmACS6* promoter in *Arabidopsis thaliana* in response to waterlogging stress. **B** Schematic of predicted ERF binding sites in the *CmACS6* promoter. **C** Yeast one-hybrid assay demonstrating the binding of CmHRE2-like to *CmACS6* promoter. **D** EMSA assay showing direct binding of CmHRE2-like protein to the *CmACS6* promoter in vitro. **E** ChIP-qPCR analysis confirming CmHRE2-like directly binding to the *CmACS6* promoter. **F** Luciferase reporter analysis indicating CmHRE2-like activates the expression of *CmACS6* in tobacco. **G** Dual-luciferase assay demonstrating CmHRE2-like stimulates *CmACS6* promoter activity in chrysanthemum protoplasts. Error bars represent standard deviation (SD, *n* = 3); Different lowercase letters indicate significant differences at *P* < 0.05 (Student's t-test and ANOVA, Turkey's correction)

To further examine the expression specificity of the *CmACS6* gene, we generated transgenic Arabidopsis plants harboring a 1,139 bp promoter fragment of the *CmACS6* gene fused to the *β-glucuronidase* gene (*CmACS6pro:GUS*). GUS staining was performed following waterlogging treatment, and the results revealed enhanced GUS signals in leaves and roots post-waterlogging, indicating that the activity of the *CmACS6* promoter was increased after waterlogging (Fig. 4A).

Given the significant yet unexplored relationship between ethylene and chrysanthemum under waterlogging conditions, we employed three predicted ERF binding sites (Fig. 4B, cis-elements 1/2/3 predicted by PlantRegMap, Table S2) of the *CmACS6pro* as probes to identify upstream transcription factors using DNA affinity trapping (Figure S6, Table S3) (Gabrielsen et al. 1989; Hu et al. 2016). The mass spectrometry results revealed numerous upstream transcription factors annotated as ERFs, one of which had been previously investigated (Wang et al. 2024). We selected *CmHRE2-like* for further validation of its binding to *CmACS6pro*.

Yeast one-hybrid analysis was conducted to examine whether CmHRE2-like directly regulates *CmACS6*. All *CmACS6* promoter baits with pGADT7-CmHRE2-like and pGADT7-GUS exhibited robust growth on SD/-T/-H/-L (SD/-Trp-His-Leu) medium. Yeast cells containing the *CmACS6* promoter transformed with pGADT7-CmHRE2-like grew on SD/-T/-H/-L medium supplemented with 50 mM 3-AT. In contrast, all *CmACS6* promoter baits with pGADT7-GUS (the negative control) failed to grow on SD/-T/-H/-L medium supplemented with 50 mM 3-AT (Fig. 4C). Subsequently, electrophoretic mobility shift assay (EMSA) was employed to determine whether CmHRE2-like could directly bind to the *CmACS6* promoter in vitro. The results demonstrated that CmHRE2-like- GST, but not GST alone, firmly bound to the biotin-labeled probes (Biotin-P) containing cis-element 3 (GCC-like). However, mutations in cis-element 3 (Biotin-mP) completely abolished CmHRE2-like-GST binding to the probe. The other unlabeled probes competed for probe binding in a dose-dependent manner (Fig. 4D). These findings suggest that CmHRE2-like directly binds to the *CmACS6* promoter in vitro.

To further validate the binding of CmHRE2-like to the *CmACS6* promoter in vivo, chromatin immunoprecipitation (ChIP)-qPCR analysis was conducted. The results demonstrated that the 'P3' region of the *CmACS6* promoter, containing 'cis-element 3 (GCC-like motif)', exhibited significant enrichment in the *CmHRE2-like* overexpression lines (35S::CmHRE2-like-GFP). Conversely, no enrichment was observed in the 'P1', 'P2', and 'CK' regions of the *CmACS6* promoter (Fig. 4E). These findings collectively indicate that CmHRE2-like directly activates *CmACS6* expression by binding to its promoter via the GCC-like motif.

We further employed the LUC system to investigate the binding of CmHRE2-like to the *CmACS6* promoter in vivo using a tobacco transient expression LUC assay (Fig. 4F) and a chrysanthemum transient expression dual-luciferase assay (Fig. 4G). The results demonstrated that the upstream transcription factor CmHRE2-like significantly activated the *CmACS6* promoter expression in both tobacco and chrysanthemum protoplasts, indicating that CmHRE2-like can activate *CmACS6* expression in vivo.

### CmHRE2-like plays a negative role in regulating waterlogging tolerance in chrysanthemum

**Fig. 5:**
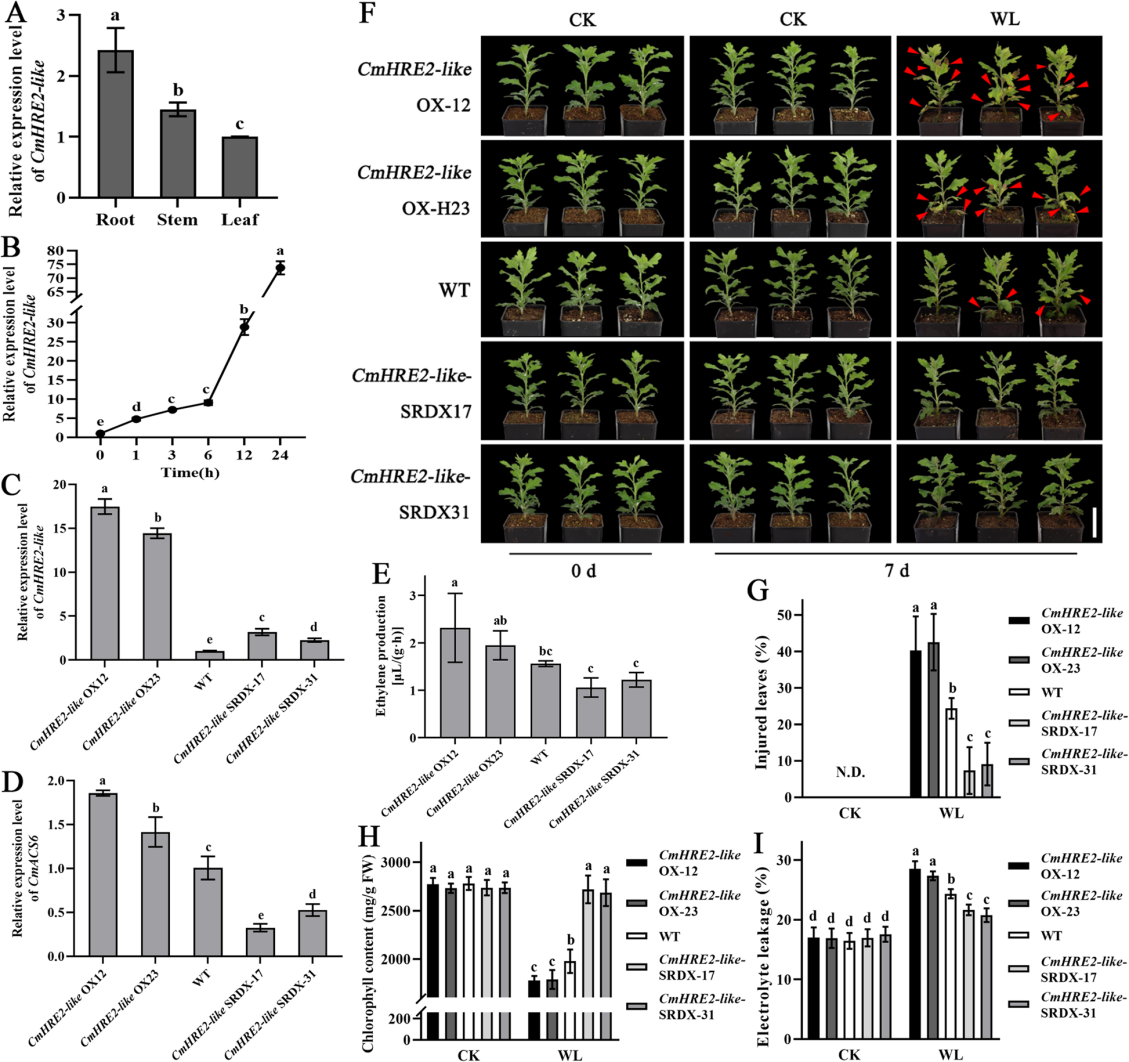
Phenotype of chrysanthemum *CmHRE2-like* overexpression and silencing plants. **A** The expression pattern of *CmHRE2-like* gene in different tissues of chrysanthemum ‘Jinba’. **B** Expression pattern of *CmHRE2-like* in response to waterlogging stress. **C** *CmHRE2-like* expression levels in *CmHRE2-like* transgenic lines. **D** *CmACS6* expression levels in *CmHRE2-like* transgenic lines. **E** Ethylene production of *CmHRE2-like* transgenic lines and WT. **F** Phenotype of *CmHRE2-like* transgenic plants under natural growth conditions (0 d) and waterlogging (7 d) conditions. Red arrows indicate the injured leaves after waterlogging stress. Bar: 5 cm. **G-I** Physiological index determinations of *CmHRE2-like* transgenic and wild-type chrysanthemum under natural growth conditions (CK) and WL. **G** Percentage of injured leaves. N.D.: Not detected. **H** Chlorophyll content. **I** Electrolyte leakage. Error bars represent standard deviation (SD, *n* = 3). Lowercase letters indicate significant differences at *P* < 0.05 (ANOVA, Tukey’s correction)

The *CmHRE2-like* gene exhibits high expression in chrysanthemum root (Fig. 5A) and is significantly upregulated following waterlogging stress (Fig. 5B). We investigated the expression level of the downstream *CmACS6* gene in the CmHRE2-like transgenic lines (Fig. 5C). The findings revealed that the expression level of the *CmACS6* gene was significantly upregulated in the *CmHRE2-like* overexpression lines (*CmHRE2-like*-OX) and considerably downregulated in *CmHRE2-like* silencing lines (*CmHRE2-like*-SRDX) (Fig. 5D). Consistent with this observation, ethylene production in *CmHRE2-like*-OX lines exceeded that of *CmHRE2-like*-SRDX lines (Fig. 5E), suggesting that the *CmHRE2-like* gene positively regulates ethylene biosynthesis.

To further investigate the role of *CmHRE2-like* in the waterlogging stress response of chrysanthemums, we subjected both *CmHRE2-like* transgenic lines and wild-type lines to waterlogging stress treatment and observed their phenotypes. After 7 days of waterlogging treatment, *CmHRE2-like*-OX lines displayed waterlogging stress-sensitive characteristics, exhibiting more etiolated and wilted leaves compared to WT (Fig. 5F). The *CmHRE2-like*-OX lines showed a significant increase in the percentage of injured leaves and electrolyte leakage (Fig. 5G-H), as well as a more substantial decrease in chlorophyll content compared to both WT and *CmHRE2-like*-SRDX lines (Fig. 5I). These observations indicate severe damage in the *CmHRE2-like*-OX lines during waterlogging treatment, while the *CmHRE2-like*-SRDX lines demonstrated enhanced waterlogging tolerance. These findings suggest that the *CmHRE2-like* gene negatively regulates waterlogging tolerance in chrysanthemum.

### *CmACS6* is required for CmHRE2-like mediated ethylene synthesis and waterlogging response

**Fig. 6:**
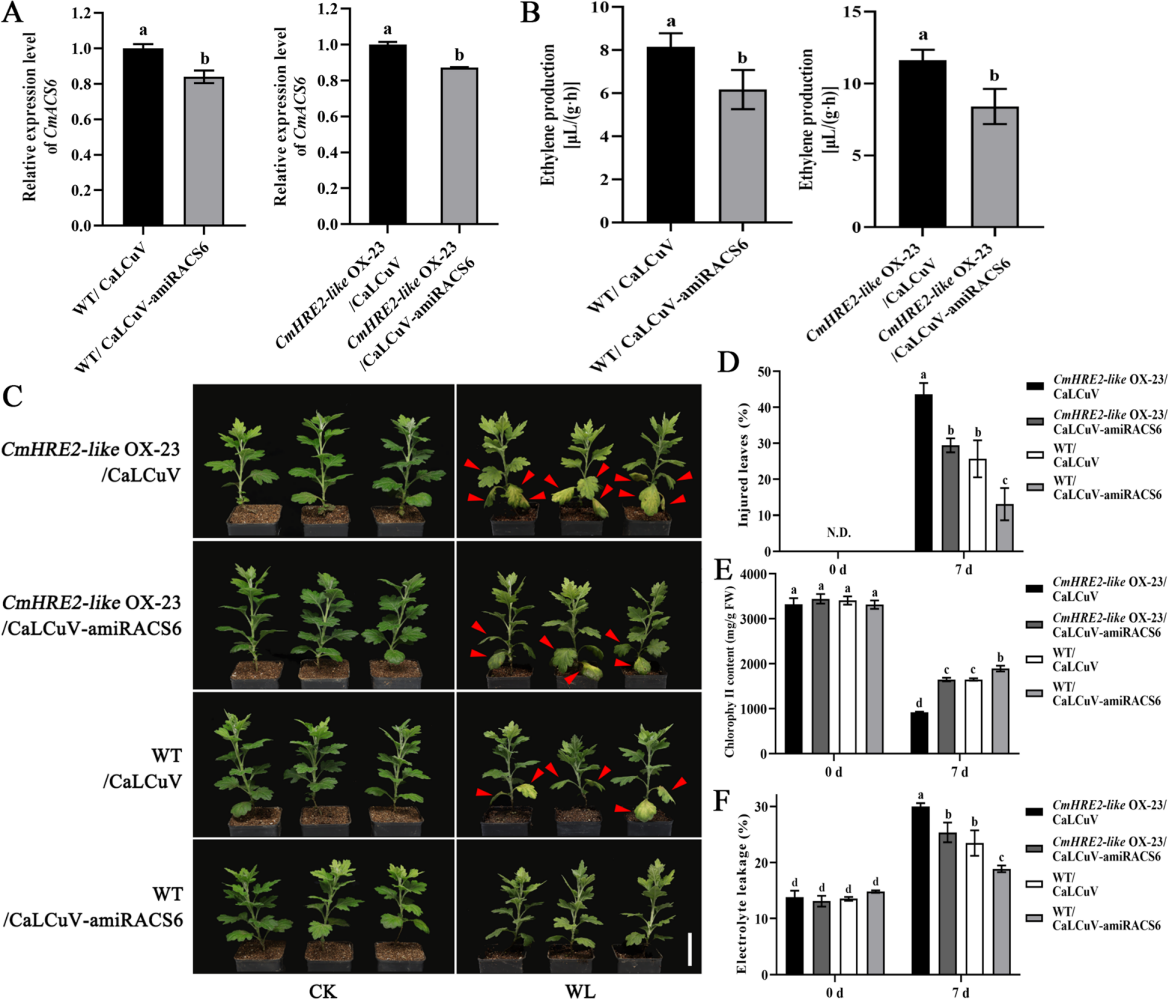
CmHRE2-like exhibits genetic effects upstream of *CmACS6* in chrysanthemum. **A** Expression levels of the *CmACS6* gene in WT-vector, WT-amiRACS6, *CmHRE2-like*-vector, and *CmHRE2-like*-amiRACS6 transgenic lines. **B** Ethylene production in transgenic lines. **C** Phenotype of *CmHRE2-like* transgenic plants under natural growth CK and waterlogging (WL) conditions. Red arrows indicate injured leaves after waterlogging stress. Bar: 5 cm. (d**-**f) Physiological index determinations of *CmHRE2-like* transgenic and wild-type chrysanthemum under natural growth CK and WL. **D** Percentage of injured leaves. N.D.: Not detected. **E** Chlorophyll content. **F** Electrolyte leakage. Error bars represent standard deviation (SD, *n* = 3). Lowercase letters indicate significant differences at *P* < 0.05 (Student's t-test and ANOVA, Tukey's correction)

To further verify whether *CmHRE2-like* regulates ethylene biosynthesis and response to waterlogging in a *CmACS6*-dependent manner, we silenced *CmACS6* in *CmHRE2-like* OX-23 lines via CaLCuV-amiRACS6 inoculation. The results indicate that the expression of the *CmACS6* gene in *CmHRE2-like* OX-23/CaLCuV-amiRACS6 infected (*CmHRE2-like*-amiRACS6) lines was marginally lower than that observed in *CmHRE2-like* OX-23/CaLCuV vector infected (*CmHRE2-like*-vector) lines (Fig. 6A). Furthermore, Fig. 6B demonstrates that *CmHRE2-like*-amiRACS6 lines produced less ethylene compared to *CmHRE2-like*-vector lines.

To assess chrysanthemum's performance under waterlogging stress following transient silencing of the *CmACS6* gene in the *CmHRE2-like* OX-23 and wild type, four transgenic lines underwent waterlogging stress treatment: WT/CaLCuV (WT-vector), WT/CaLCuV-amiRACS6 (WT-amiRACS6), *CmHRE2-like*-vector, and *CmHRE2-like*-amiRACS6. After 6 days of waterlogging treatment, the *CmHRE2-like*-amiRACS6 lines demonstrated reduced waterlogging injury symptoms compared to the *CmHRE2-like*-vector lines (Fig. 6C). Moreover, the *CmHRE2-like*-amiRACS6 lines exhibited a lower percentage of injured leaves and electrolyte leakage, along with higher chlorophyll content (Fig. 6D-F). These findings suggest that *CmHRE2-like*-amiRACS6 lines possess slightly improved tolerance to waterlogging stress due to the down-regulation of the *CmACS6* gene. Collectively, these results indicate that the regulation of chrysanthemum's response to waterlogging stress by CmHRE2-like is partially dependent on *CmACS6*.

## Discussion

Several studies have indicated that leaf etiolation and senescence are stress-adaptive phenotypes in plants (Schippers et al. 2015). However, in chrysanthemum cultivation, severe leaf etiolation and senescence not only inhibit plant growth but also diminish ornamental value. Consequently, leaf health is considered a crucial indicator of chrysanthemum's tolerance to waterlogging conditions. In previous waterlogging stress pre-tests, chrysanthemum 'Jinba' exhibited minimal adventitious root production during early waterlogging stages. Adventitious root formation was observed only after three to four weeks of continuous waterlogging. By this time, the above-ground portions of the chrysanthemum plants had sustained significant damage, displaying severely yellowed, decayed, and wilted leaves (data not shown), resulting in a loss of ornamental value. Therefore, it is hypothesized that the time required for ethylene-induced adventitious root production in chrysanthemum 'Jinba' is too prolonged to induce near-term waterlogging tolerance. Concurrently, substantial ACC accumulation was observed in the roots under waterlogging stress conditions following rapid up-regulation of *CmACS*s gene expression, with ACC being transported upward from the roots. In the oxygen-rich region near the water surface, ACC was ultimately oxidized to ethylene. This explains why ethylene production within chrysanthemum roots did not increase following a 1-h waterlogging period, in contrast to sustained elevated ethylene levels in leaves (Fig. 1A and B). The massive ethylene burst induces yellowing of plant leaves near the water surface (Figs. 1, and 2). Significant up-regulation of chlorophyll metabolism-related genes and leaf senescence genes was detected in *CmACS6* overexpressing plants (Fig. 3), leading to the hypothesis that leaf chlorosis under waterlogging stress might be due to chlorophyll metabolism alterations. Recent evidence from grape (*Vitis vinifera* L.) indicates that elevated ethylene levels can reduce chlorophyll content by regulating chlorophyll degradation pathways (Li et al. 2023b). Future research will focus on the specific advantageous traits of the waterlogging-tolerant cultivar 'Nannong Xuefeng' once stable transformation lines are successfully created.

Nevertheless, the phenotypic differences between *CmACS6*-silenced lines and wild-type chrysanthemums were not notably pronounced following waterlogging exposure. This observation may be attributed to the functional redundancy within the relatively conserved ACS gene family in chrysanthemums. During waterlogging stress, other ACS homologs responsive to such conditions (e.g., *CmACS1* and *CmACS7*) potentially compensated for the partial loss of *CmACS6* function (Zhao et al. 2018), thereby mitigating the expected phenotypic effects.

Ethylene serves as a crucial regulator under both abiotic and biotic stress conditions, responding rapidly to adversities such as hypoxia, waterlogging, heat, cold, salt stress, drought, injury, and pathogen attack. Plants respond to stress by modulating ethylene production (Argueso et al. 2007). This is typically achieved by enhancing ACS activity to increase ACC production, resulting in elevated ethylene levels (Adams and Yang 1979; Zhou et al. 2020). Ethylene has long been recognized as a positive regulator of plant adaptation to hypoxia and waterlogging tolerance by inducing adaptive phenotypes such as the production of adventitious roots and aerated tissues (Liu et al. 2022). However, it has also been suggested that excessive ethylene accumulation during waterlogging may induce a negative response and reduce the plant's waterlogging tolerance. *SUB1A*, a key gene for flood tolerance in deepwater rice, is induced by submergence and ethylene, but SUB1A also represses ethylene production during submergence through feedback regulation (Fukao et al. 2006). Under waterlogging stress, the waterlogging-sensitive apple rootstock (*M. toringoides*) produced more ethylene than the waterlogging-tolerant apple rootstock (*M. hupehensis*) and exhibited more severe leaf chlorosis (Zhang et al. 2023). Additionally, ethylene's negative regulation of waterlogging stress has been reported more frequently during waterlogging stress reoxygenation. Flood-tolerant rice cultivars generally show gradual leaf chlorosis during flood recovery, which may be triggered by ethylene-induced flooding, promoting enzymatic degradation of chlorophyll and carbohydrate consumption. Blocking ethylene action with the ethylene inhibitor 1-MCP significantly increased the survival of flooded plants with reduced gene expression and enzyme activity of vegetative chlorophyllase and reduced chlorophyll degradation, as well as inhibited starch and soluble sugar consumption during flood stress. It facilitated plant recovery from flooding (Ella et al. 2003). In previous studies with Valencia orange (*Citrus sinensis cv.* Valencia), exogenously applied ethylene accelerated chlorophyll degradation by increasing de novo synthesis of chlorophyllase (Jacob Wilk et al. 1999; Trebitsh et al. 1993). Studies on the *Arabidopsis* germplasm Bay-0 and Lp2-6 revealed that ethylene plays a negative regulatory role in recovery from waterlogging stress. Lp2-6, which is more tolerant to waterlogging, reduced plant ethylene production by repressing the expression of *ACS* genes, whereas more ethylene was produced in Bay-0 plants, which reopened their stomata earlier after reoxygenation and were more responsive to ABA, suggesting that ethylene may inhibit ABA-regulated stomatal closure and thus regulate stomatal opening in Bay-0. After flood reoxygenation, ethylene formed through *ACS* and *ACO* could accelerate dehydration and senescence in *Arabidopsis* after flood reoxygenation by inducing the expression of *Senescence-associated Gene 113* (*SAG113*) and transcription factor *ORESARA1* (*ORE1/NAC6*) (Yeung et al. 2018). *Arabidopsis ACS7* has been reported to be a hub for "cross-talk" between ET and ABA and to negatively regulate ABA biosynthesis, and acs7 mutants have enhanced tolerance to salt, heat, and osmotic stress (Dong et al. 2011). In this study, we found that chrysanthemum 'Jinba' exhibited a more sensitive phenotype to waterlogging stress with significant leaf chlorosis after exogenous ethylene treatment (Fig. 1C), suggesting that elevated ethylene leads to poorer waterlogging tolerance in chrysanthemum 'Jinba'. We cloned the gene *CmACS6*, which encodes ACC synthase, a key rate-limiting enzyme for ethylene biosynthesis, in chrysanthemum 'Jinba' (Figure S2A), and characterized ethylene production and waterlogging tolerance of the transgenic lines after overexpression and disruption of the gene in 'Jinba'. Compared with the wild type, the *CmACS6*-OX lines exhibited significantly higher ethylene production and significantly poorer waterlogging tolerance, with a higher percentage of damaged leaves, lower leaf chlorophyll content, and higher levels of leaf ion leakage (Fig. 2D-G). In contrast, ethylene production and waterlogging tolerance in the *CmACS6* interference lines were diametrically opposite to those in the overexpression lines, suggesting that the *CmACS6* gene negatively regulates waterlogging tolerance in chrysanthemum 'Jinba'. The results were consistent with the phenotypes of overexpression of *AtACS7* and *acs7* mutants in *Arabidopsis*, where *AtACS7* overexpression lines showed significantly increased ethylene levels and enhanced plant sensitivity to flooding, whereas *acs7* mutants showed considerably reduced ethylene production and enhanced flooding tolerance under flooding stress (Zhang et al. 2023).

**Fig. 7:**
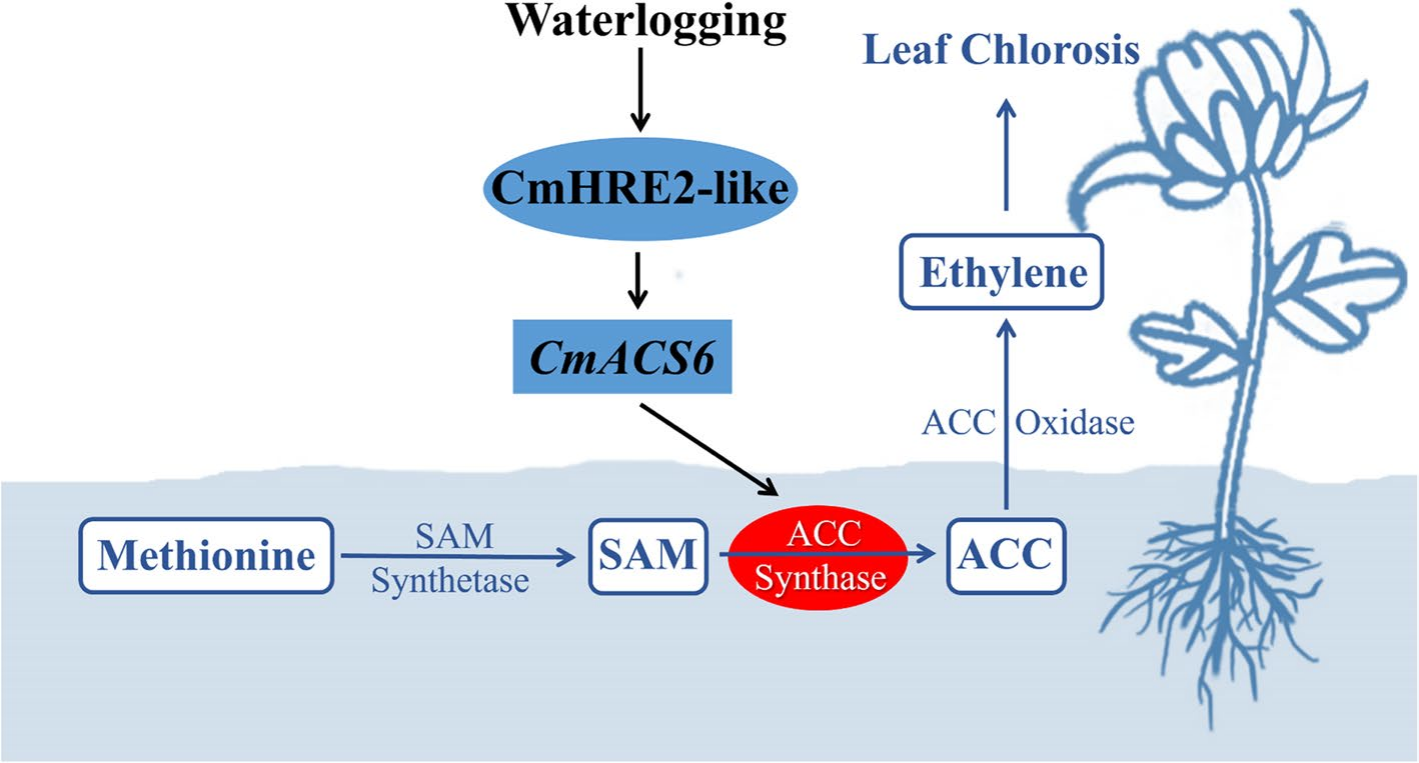
Molecular mechanism of CmHRE2-like-CmACS6 regulation of ethylene biosynthesis in response to waterlogging stress in chrysanthemum

This study has identified a novel molecular module, CmHRE2-like-CmACS6, which regulates ethylene biosynthesis and waterlogging tolerance in chrysanthemums (Fig. 7). *CmACS6* functions downstream of *CmHRE2-like* in both ethylene biosynthesis and waterlogging response. The regulation of chrysanthemum's response to waterlogging stress by CmHRE2-like is partially dependent on the *CmACS6* gene. CmHRE2-like negatively regulates chrysanthemum waterlogging tolerance by promoting the transcription of the *CmACS6* gene, which in turn increases ethylene production in the plant. This elevated ethylene level causes chrysanthemums to exhibit leaf chlorosis under waterlogging stress.

## Methods

### Plant materials and treatment

This study employs the common commercial chrysanthemum cultivar 'Jinba' for all experimental treatments. This cultivar was developed at the Chrysanthemum Germplasm Resource Preserving Center, situated at Nanjing Agricultural University, China. The chrysanthemum plants were cultivated in a growth chamber under controlled conditions of 25 °C: 18 °C, with a 16 h: 8 h day: night cycle.

To analyze the gene expression profiles in response to waterlogging stress, the second to fourth leaves near the stem base were collected at various time points (0 h, 1 h, 3 h, 6 h, 9 h, 12 h, and 24 h) following waterlogging treatment for RNA isolation. The experiment was conducted in triplicate, with each sample comprising three individual plant replicates per time point. To determine gene expression in different tissues, root, stem, and leaf samples were isolated from chrysanthemums with 10 to 12 leaves for further analysis. This experiment was also repeated three times, with each sample containing three individual plant replicates.

For waterlogging treatments, plants were placed in plastic containers with the water level maintained 4–5 cm above the soil surface (Su et al. 2016). To mitigate the impact of circadian rhythms, chrysanthemum plants were subjected to waterlogging at staggered intervals, with all treatments concluding and sample collection occurring simultaneously. The control group (0 h) remained untreated and was sampled concurrently (Su et al. 2016; Yang and Wei 2015; Su 2019).

Prior to waterlogging stress treatment, plants underwent pretreatment with an ethylene solution (100 mg/L). Each chrysanthemum specimen received 5 mL of ethylene solution via root irrigation, followed by a 6-h interval before conventional waterlogging stress treatment. The group subjected solely to ethylene treatment received conventional watering subsequent to the pretreatment phase.

### Quantitative real-time PCR (RT-qPCR)

Relative expression was quantified using RT-qPCR. Total RNA was extracted and reverse transcribed, and relative expression was determined by RT-qPCR using SYBR Premix Ex Taq TM II (Tli RNaseH Plus; Takara). Data analysis employed the −2^ΔΔC^_T_ method. The chrysanthemum *CmEF1α* (KF305681) served as the reference gene. Each RT-qPCR experiment included three biological and technical replicates. The primers utilized are listed in Table S1.

### Measurement of electrolyte leakage

Electrolyte leakage was evaluated in plants subjected to waterlogging stress and control conditions, following the methodology described by Tang et al., (2021). In brief, leaf samples were obtained using a 6 mm puncher to collect small circular sections from all plants. These sections were placed in tubes containing 5 mL of deionized water. After 3–4 h of agitation at room temperature, the initial electrical conductivity was measured (denoted as A). Subsequently, the solution was heated to boiling for 30 min, and the electrical conductivity was reassessed (denoted as B). The ratio A/B was then calculated to quantify electrolyte leakage.

### Measurement of injured leaves ratio

Following exposure to waterlogging stress, plant morphology was examined and the percentage of injured leaves was quantified. Under waterlogging conditions, chrysanthemum leaves typically exhibit waterlogging-sensitive phenotypes, including yellowing, browning, rotting, drying, and wilting. Leaves displaying these characteristics are collectively categorized as injured leaves. The injured leaves ratio is calculated using the following formula: [number of damaged leaves (exhibiting yellowing, browning, rotting, drying, or wilting)/total number of mature leaves on chrysanthemum plants] × 100%.

### Measurement of chlorophyll content

The second to fourth leaves proximal to the stem base were harvested for chlorophyll content analysis. Leaf fragments were immersed in 95% ethyl alcohol and kept in darkness for 24 h. Subsequently, absorbance measurements at 649 nm and 665 nm were obtained using a microplate reader. The specific methodology and calculations followed the protocol delineated by Gao et al. (2021).

### Determination of ethylene production

Samples were collected in 20 mL sealed glass vials and allowed to settle at 25℃ for 12 h to ensure complete ethylene evaporation. The glass vials were shaken several times, after which 1 mL of gas sample was extracted from the headspace using a gas-tight syringe and injected into a gas chromatograph (GC9790Plus, Zhejiang, China) equipped with an Al_2_O_3_/S column. The column, injector, and GC-FID temperatures were set at 80℃, 220℃, and 250℃, respectively, with the carrier gas N_2_ flow rate maintained at 3 mL/min. The ethylene production rate of chrysanthemum was calculated using the formula (µL·g^−1^·h^−1^): (c × V)/(m × t), where c represents the ethylene content of the sample (µL/L), V denotes the glass container volume (mL), t indicates the sample standing time (h), and m indicates the sample weight (g). The experiment was conducted with six biological replicates for each group (Wang et al. 2022).

### Gene isolation and sequence analysis

The complete ORF sequence of *CmACS6* was isolated from chrysanthemum 'Jinba' using the primer pair CmACS6-F/-R (see Table S1), employing reverse transcription amplification as the template. The sequence was subsequently inserted into the pMD19-T vector (Takara Bio) and verified through sequencing. Multiple sequence alignments of CmACS6 protein homologs, obtained from the National Center for Biotechnology Information (NCBI) database (https://www.ncbi.nlm.nih.gov/), were conducted using DNAMAN software. A neighbor-joining phylogenetic tree was constructed using MEGA 5.02 software with 1,000 bootstrap replications. Detailed information regarding CmACS6 is presented in Figure S1.

### Genetic transformation of chrysanthemum

To generate transgenic lines, the pORE-R4-*CmACS6* and pORE-2 × 35SAA-amiR*CmACS6* plasmids were introduced into *Agrobacterium tumefaciens* strain EHA105. Subsequently, the chrysanthemum cultivar 'Jinba' underwent transformation using the Agrobacterium-mediated leaf disc method, as previously described (Li et al. 2023a). DNA from transgenic lines was extracted using a Rapid Plant Genomic DNA Isolation Kit (Sangon Biotech, Shanghai, China), and positive plants were identified using 35S-F/gene-R primers (Table S1). Confirmed transgenic plants were transferred from the culture medium to the greenhouse for standard cultivation practices.

The Web MicroRNA Designer (http://wmd3.weigelworld.org/cgi-bin/webapp.cgi) was utilized to design the 21-nucleotide mature amiRNA-*CmACS6* sequence and primers for the amplification of amiR-*CmACS6*.

### ACC synthase activity

To assess ACC synthase activity in vitro, the ORF of CmACS6 was cloned into the pET32a vector and subsequently transformed into Escherichia coli strain BL21. The resulting HIS-CmACS6 fusion protein was utilized for the ACC synthase activity assay. The quantification of ACC content followed the methodology described by Lizada and Yang (1979).

To assess ACC synthase activity in vivo, leaf samples were collected from transgenic lines and wild-type chrysanthemum. The comprehensive methodology has been previously detailed in studies by Boller et al. (1979) and Zhu et al. (2006). The quantification of ACC content follows the protocol established by Lizada and Yang (1979).

### Virus-induced gene silencing

To suppress *CmACS6* expression in *CmHRE2-like* transgenic lines and wild-type 'Jinba', a virus-based microRNA expression system was utilized, following the method previously described by Tang et al. (2010). The amiR-*CmACS6* sequence was inserted into the pCVA vector (CaLCuV-amiRACS6) and subsequently introduced into *Agrobacterium tumefaciens* strain GV3101. Experimental procedures were conducted as outlined by Li et al. (2023a). The vector combinations employed were CaLCuV-amiRACS6 + pCVB and pCVA + pCVB, with pCVA and pCVB serving as empty vectors. Thirty-day-old chrysanthemum plants, including both *CmHRE2-like* OX-23 and WT, cultivated on MS medium, were subjected to infiltration.

### RNA extraction, transcriptome sequencing and bioinformatic analysis

Leaf samples (the second to fourth leaves near the stem base) were collected from 10- to 12-leaf-old transgenic lines (*CmACS6* OX-12 and *CmACS6* amiR-7) and wild-type (WT) plants. Each sample comprised three biological replicates. RNA extraction was performed using the Plant RNA Isolation Kit (Waryong, Beijing, China) according to the manufacturer's instructions. Following rigorous quality assessment, the extracted RNA underwent sequencing using an Illumina HiSeq™2000 instrument at the Beijing Genomics Institute (BGI, http://www.genomics.cn/index; Shenzhen, China). Gene Ontology (GO) and KEGG (Kyoto Encyclopedia of Genes and Genomes) pathway enrichment analyses of the annotated DEGs were conducted using the Beijing Genomics Institute (BGI) Interactive Reporting System (https://report.bgi.com/ps/login/login.html). Heatmaps were generated using TBtools-II (v2.019) (Chen et al. 2020) to visualize the data.

### GUS staining

The promoters of *CmACS6* were inserted into pCAMBIA1301 and introduced into *Agrobacterium tumefaciens* strain (GV3101). The resulting construct was then used to transform Arabidopsis via the pollen tube channel method. Transgenic lines, both with and without waterlogging treatment, were subjected to GUS staining experiments. These experiments were conducted following the protocol provided in the Gusblue Kit (Waryong, Beijing, China). The primer sets used for all constructs are detailed in Table S1.

### Yeast one-hybrid screening (Y1H)

The ORF of *CmHRE2-like* was inserted into the pGADT7 (AD) vector, while the promoters of *CmACS6* were incorporated into pHIS2 vectors. AD-CmHRE2-like was co-transformed with pHIS2-*CmACS6pro* into yeast strain Y187. The negative control consisted of yeasts co-transformed with the pHIS2 and pGADT7-GUS plasmids. Yeast cells were incubated on medium (SD/-Trip/-His/-Leu) at 30 °C for 3 days, followed by incubation on the same SD medium supplemented with 50 mM 3-AT (3-amino-1,2,4-triazole) for 3–5 days at 30 °C. Primer sets for all constructs are provided in TableS1.

### EMSA

The complete ORF of CmHRE2-like was inserted into the pGEX4T-1 vector (Waryong, Beijing, China; Table S1) and subsequently transformed into *Escherichia coli* strain BL21. The expressed GST-CmHRE2-like protein was then purified using GST beads (Promega, Madison, WI, USA). Probes were labeled with biotin utilizing the EMSA Probe Biotin Labeling Kit (Beyotime, Shanghai, China), with primer sequences provided in Table S1. The interaction between GST-CmHRE2-like protein and *CmACS6pro* was confirmed using the LightShift Chemiluminescent EMSA Kit (Thermo Fisher Scientific, Waltham, USA).

### ChIP-PCR assays

The ChIP assay was conducted utilizing the chrysanthemum *CmHRE2-like* overexpression line. Chromatin complexes were extracted from leaves of 4-week-old chrysanthemum tissue culture plants, fixed in 1% formaldehyde, and rapidly frozen in liquid nitrogen. The isolation procedure adhered to the method outlined by Li et al*.* ( 2023a). Quantitative real-time PCR analysis was performed using primers designed to amplify the *CmACS6* promoter region (Table S1). The experiment incorporated three biological replicates and three technical replicates for each primer. 35S::GFP plants served as controls, with an equivalent number of biological and technical replicates.

### Luciferase assays

A luciferase assay was employed to assess the capability of CmHRE2-like to activate the CmACS6 promoter in tobacco leaves and chrysanthemum protoplasts.

To conduct the luciferase assay, a 1,139 bp promoter fragment of the *CmACS6* promoter was inserted into the transient expression vector pCAMBIA1381Z-LUC as a reporter. Additionally, the coding sequence of *CmHRE2-like* was cloned into pMDC43, driven by the 35S promoter, as an effector. Appropriate combinations of transformed Agrobacteria were mixed and co-infiltrated into the epidermal cells of *Nicotiana benthamiana* leaves. The plants were then incubated at 22 °C for 48 h. Subsequently, the transiently transformed tobacco leaves were sprayed with D-luciferin Sodium Salt (Solarbio, Beijing, China) and examined under a Tanon 5200 multi-imaging apparatus (Tanon, Shanghai, China) to observe the luciferase activity in *N. benthamiana* leaves co-transformed with different plasmid combinations. Primer sets for all constructs are listed in Table S1.

For the dual-luciferase assay, a 1,139 bp promoter fragment of the CmACS6 promoter was inserted into the transient expression vector pGreenII0800-LUC. The complete ORF sequence of CmHRE2-like was cloned into pMDC43, driven by the 35S promoter. These constructs were co-expressed in chrysanthemum protoplasts as described previously (Yoo et al. 2007). The Dual-Glo Luciferase Assay System (Promega, Beijing, China) was used to examine LUC expressions. The LUC/REN ratio was measured using the Dual-Glo Luciferase Assay System (Promega, Beijing, China) with an Infinite M200 luminometer (Tecan, Männedorf, Switzerland). All transient transformations were performed using three biological replicates. Primer sets for all constructs are listed in Table S1.

### Statistical analysis

Statistical analyses were conducted using SPSS v25.0 software (SPSS Inc., Chicago, IL, USA). The ANOVA-Tukey correction test was employed to assess statistical significance across different transgenic lines, tissues, and phenotypic data following waterlogging at *P* < 0.05. Additionally, Student's t-test was utilized to evaluate significant differences in relative expression levels and LUC/REN ratios between two groups. Significance levels were denoted using different lowercase letters.

## Supplementary Information

Supplementary Material 1: Figure S1 Heatmap analysis of DEGs in 'Qinglu' and 'Nannong Xuefeng' related to ACS genes (Zhao* et al.*,^2018^). Figure S2 Cloning of* CmACS6* and characterization of CmACS6 protein in chrysanthemum. Figure S3 Identification of *CmACS6* transgenic lines. Figure S4 The performance of *CmACS6* transgenic lines under inundation conditions. Figure S5 The root system of CmACS6 transgenic lines after waterlogging. Figure S6 SDS-PAGE analysis of DNA affinity trapping.

Supplementary Material 2: Table S1 Primer sequences used in this study. Table S2 Prediction of CmACS6pro by PlantRegMap. Table S3 Results of DNA affinity trapping.

## Data Availability

All data generated or analyzed during this study are included in this article and the supplementary information files.

## Abbreviations

3-AT: 3-Amino-1,2,4-triazole
ACC: 1-Aminocyclopropane-1-carboxylic acid
ACS: 1-Aminocyclopropane-1-carboxylic acid synthase
CaLCuV: Cabbage leaf curl virus
ChIP: Chromatin Immunoprecipitation
EMSA: Electrophoretic mobility shift assay
ERF: Ethylene responsive factor
GFP: Green fluorescent protein
GO: Gene Ontology
GUS: β-Glucuronidase
His: Histidine
HRE: Hypoxia Responsive ERF gene
KEGG: Kyoto Encyclopedia of Genes and Genomes
LUC: Luciferase assay
ORF: Open reading frame
OX: Over expression
qRT-PCR: Quantitative real-time PCR
REN: Renilla luciferase
RNA-seq: RNA sequencing
ROS: Reactive oxygen species
SD: Synthetic dropout medium
WL: Waterlogging
WT: Wild type
Y1H: Yeast one-hybrid

## References

[CR1] Adams DO, Yang SF. Ethylene biosynthesis: Identification of 1-aminocyclopropane-1-carboxylic acid as an intermediate in the conversion of methionine to ethylene. P Natl Acad Sci Usa. 1979;76:170–4. 10.1073/pnas.76.1.170 PMC382898 16592605

[CR2] Ahsan N, Lee DG, Lee SH, Kang KY, Bahk JD, Choi MS, Lee IJ, Renaut J, Lee BH. A comparative proteomic analysis of tomato leaves in response to waterlogging stress. Physiol Plantarum. 2007;131:555–70. 10.1111/j.1399-3054.2007.00980.x 18251847

[CR3] Argueso CT, Hansen M, Kieber JJ. Regulation of ethylene biosynthesis. J Plant Growth Regul. 2007;26:92–105.

[CR4] Boller TS, Herner RRC and Kende H. Assay for and Enzymatic Formation of an Ethylene Precursor, 1-Aminocyclopropane-l-Carboxylic Acid. Planta. 1979;145(3)293–303. 10.1007/BF00454455. 10.1007/BF00454455 24317737

[CR5] Chen C, Chen H, Zhang Y, Thomas HR, Frank MH, He Y, Xia R. TBtools: An Integrative Toolkit Developed for Interactive Analyses of Big Biological Data. Mol Plant. 2020;13:1194–202. 32585190 10.1016/j.molp.2020.06.009

[CR6] Dawood T, Yang XP, Visser E, Te Beek T, Kensche PR, Cristescu SM, Lee S, Flokova K, Nguyen D, Mariani C, Rieu I. A Co-opted hormonal cascade activates dormant adventitious root primordia upon flooding in Solanum dulcamara. Plant Physiol. 2016;170:2351–64. 26850278 10.1104/pp.15.00773 PMC4825138

[CR7] Dong H, Zhen ZQ, Peng JY, Chang L, Gong QQ, Wang NN. Loss of ACS7 confers abiotic stress tolerance by modulating ABA sensitivity and accumulation in Arabidopsis. J Exp Bot. 2011;62:4875–87. 21765163 10.1093/jxb/err143 PMC3193000

[CR8] Ella ES, Kawano N, Yamauchi Y, Tanaka K, Ismail AM. Blocking ethylene perception enhances flooding tolerance in rice seedlings. Funct Plant Biol. 2003;30:813–9. 32689065 10.1071/FP03049

[CR9] Eun H, Ali S, Jung H, Kim K and Kim W. Profiling of ACC synthase gene (*ACS11*) expression in *Arabidopsis *induced by abiotic stresses. Appl Biol Chem. 2019;62:42. 10.1186/s13765-019-0450-4.

[CR10] Fukao T, Xu KN, Ronald PC, Bailey-Serres J. A variable cluster of ethylene response factor-like genes regulate metabolic and developmental acclimation responses to submergence in rice(W). Plant Cell. 2006;18:2021–34. 16816135 10.1105/tpc.106.043000 PMC1533987

[CR11] Gabrielsen OS, Hornes E, Korsnes L, Ruet A, Oyen TB. Magnetic DNA affinity purification of yeast transcription factor tau–a new purification principle for the ultrarapid isolation of near homogeneous factor. Nucleic Acids Res. 1989;17:6253–67. 2671937 10.1093/nar/17.15.6253 PMC318276

[CR12] Gao W, Zhang L, Wang J, Liu Z, Zhang Y, Xue C, Liu M and Zhao J. ZjSEP3 modulates flowering time by regulating the LHY promoter. Bmc Plant Biol. 2021;21:527. 10.1186/s12870-021-03305-x. 10.1186/s12870-021-03305-x PMC8582215 34763664

[CR13] Hartman S, Liu ZG, van Veen H, Vicente J, Reinen E, Martopawiro S, Zhang HT, van Dongen N, Bosman F, Bassel GW, Visser E, Bailey-Serres J, Theodoulou FL, Hebelstrup KH, Gibbs DJ, Holdsworth MJ, Sasidharan R and Voesenek L. Ethylene-mediated nitric oxide depletion pre-adapts plants to hypoxia stress. Nat Commun. 2019;10:4020. 10.1038/s41467-019-12045-4. 10.1038/s41467-019-12045-4 PMC6728379 31488841

[CR14] Hu D, Sun C, Ma Q, You C, Cheng L, Hao Y. MdMYB1 Regulates Anthocyanin and Malate Accumulation by Directly Facilitating Their Transport into Vacuoles in Apples. Plant Physiol. 2016;170:1315–30. 26637549 10.1104/pp.15.01333 PMC4775115

[CR15] Jacob Wilk D, Holland D, Goldschmidt EE, Riov J, Eyal Y. Chlorophyll breakdown by chlorophyllase: isolation and functional expression of the Chlase1 gene from ethylene-treated Citrus fruit and its regulation during development. The Plant Journal: for Cell and Molecular Biology. 1999;20:653–61. 10652137 10.1046/j.1365-313x.1999.00637.x

[CR16] Kuai J, Zhou Z, Wang Y, Meng Y, Chen B, Zhao W. The effects of short-term waterlogging on the lint yield and yield components of cotton with respect to boll position. Eur J Agron. 2015;67:61–74.

[CR17] Li F, Zhang Y, Tian C, Wang X, Zhou L, Jiang J, Wang L, Chen F, Chen S. Molecular module ofCmMYB15-like-Cm4CL2 regulating lignin biosynthesis of chrysanthemum (Chrysanthemum morifolium) in response to aphid (Macrosiphoniella sanborni) feeding. New Phytol. 2023a;237:1776–93. 36444553 10.1111/nph.18643

[CR18] Li Z, Chen C, Zou D, Li J, Huang Y, Zheng X, Tan B, Cheng J, Wang W, Zhang L, Ye X, Feng J. Ethylene accelerates grape ripening via increasing VvERF75-induced ethylene synthesis and chlorophyll degradation. Fruit Research. 2023b;3:1–9.

[CR19] Liu ZG, Hartman S, van Veen H, Zhang HT, Leeggangers H, Martopawiro S, Bosman F, de Deugd F, Su P, Hummel M, Rankenberg T, Hassall KL, Bailey-Serres J, Theodoulou FL, Voesenek L, Sasidharan R. Ethylene augments root hypoxia tolerance via growth cessation and reactive oxygen species amelioration. Plant Physiol. 2022;190:1365–83. 35640551 10.1093/plphys/kiac245 PMC9516759

[CR20] Lizada MC, Yang SF. A simple and sensitive assay for 1-aminocyclopropane-1-carboxylic acid. Anal Biochem. 1979;100:140–5. 543532 10.1016/0003-2697(79)90123-4

[CR21] Sairam RK, Kumutha D, Ezhilmathi K. Waterlogging tolerance: nonsymbiotic haemoglobin-nitric oxide homeostasis and antioxidants. Curr Sci India. 2009;96:674–82.

[CR22] Sasidharan R, Voesenek LACJ. Ethylene-mediated acclimations to flooding stress. Plant Physiol. 2015;169:3–12. 25897003 10.1104/pp.15.00387 PMC4577390

[CR23] Schippers JHM, Schmidt R, Wagstaff C, Jing H. Living to die and dying to live: the survival strategy behind leaf senescence. Plant Physiol. 2015;169:914–30. 26276844 10.1104/pp.15.00498 PMC4587445

[CR24] Steffens B, Rasmussen A. The Physiology of Adventitious Roots. Plant Physiol. 2016;170:603–17. 26697895 10.1104/pp.15.01360 PMC4734560

[CR25] Su JS (2019) Genetic architecture and candidate genes for waterlogging tolerance of chrysanthemum, p 195: Nanjing Agricultural University. (in Chinese).

[CR26] Su JS, Zhang F, Li PR, Guan ZY, Fang WM, Chen FD. Genetic variation and association mapping of waterlogging tolerance in chrysanthemum. Planta. 2016;244:1241–52. 27522648 10.1007/s00425-016-2583-6

[CR27] Tang Y, Wang F, Zhao JP, Xie K, Hong YG, Liu YL. Virus-based microrna expression for gene functional analysis in plants. Plant Physiol. 2010;153:632–41. 20388670 10.1104/pp.110.155796 PMC2879806

[CR28] Tang H, Bi H, Liu B, Lou SL, Song Y, Tong SF, Chen NN, Jiang YZ, Liu JQ, Liu HH. WRKY33 interacts with WRKY12 protein to up-regulate RAP2.2 during submergence induced hypoxia response in Arabidopsis thaliana. New Phytol. 2021;229:106–25. 33098101 10.1111/nph.17020

[CR29] Trebitsh T, Goldschmidt EE, Riov J. Ethylene induces de novo synthesis of chlorophyllase, a chlorophyll degrading enzyme, in Citrus fruit peel. Proceedings of the National Academy of Sciences - PNAS. 1993;90:9441–5. 10.1073/pnas.90.20.9441 PMC47584 11607429

[CR30] Voesenek L, Sasidharan R. Ethylene - and oxygen signalling - drive plant survival during flooding. Plant Biol. 2013;15:426–35. 23574304 10.1111/plb.12014

[CR31] Wang Y, Zhang J, Wang X, Zhang T, Zhang F, Zhang S, Li Y, Gao W, You C, Wang X and Yu K. Cellulose nanofibers extracted from natural wood improve the postharvest appearance quality of apples. Front Nutr. 2022;9:881783. 10.3389/fnut.2022.881783. 10.3389/fnut.2022.881783 PMC9136226 35634411

[CR32] Wang Y, Zhang W, Hong C, Zhai L, Wang X, Zhou L, Song A, Jiang J, Wang L, Chen F and Chen S. Chrysanthemum (Chrysanthemum morifolium) CmHRE2-like negatively regulates the resistance of chrysanthemum to the aphid (Macrosiphoniella sanborni). Bmc Plant Biol. 2024;24:76. 10.1186/s12870-024-04758-6. 10.1186/s12870-024-04758-6 PMC10823704 38281936

[CR33] Yang CP, Wei HR. Designing Microarray and RNA-Seq Experiments for Greater Systems Biology Discovery in Modern Plant Genomics. Mol Plant. 2015;8:196–206. 25680773 10.1016/j.molp.2014.11.012

[CR34] Yang C, Lu X, Ma B, Chen SY, Zhang JS. Ethylene Signaling in Rice and Arabidopsis: Conserved and Diverged Aspects. Mol Plant. 2015;8:495–505. 25732590 10.1016/j.molp.2015.01.003

[CR35] Yeung E, van Veen H, Vashisht D, Sobral Paiva AL, Hummel M, Rankenberg T, Steffens B, Steffen-Heins A, Sauter M, de Vries M, Schuurink RC, Bazin J, Bailey-Serres J, Voesenek LACJ and Sasidharan R. A stress recovery signaling network for enhanced flooding tolerance inArabidopsis thaliana. Proceedings of the National Academy of Sciences. 2018;115(26):E6085–94. 10.1073/pnas.1803841115. 10.1073/pnas.1803841115 PMC6042063 29891679

[CR36] Yin DM (2011) Evaluation on waterlogging tolerance and its mechanisms in Chrysanthemum morifolium and its related species, p 128, Nanjing: Nanjing Agricultural University. (in Chinese)

[CR37] Yin DM, Chen SM, Chen FD, Guan ZY, Fang WM. Morphological and physiological responses of two chrysanthemum cultivars differing in their tolerance to waterlogging. Environ Exp Bot. 2009;67:87–93.

[CR38] Yin J, Niu L, Li Y, Song X, Ottosen C, Wu Z, Jiang F and Zhou R. The effects of waterlogging stress on plant morphology, leaf physiology and fruit yield in six tomato genotypes at anthesis stage. Vegetable Research. 2023;3:31. 10.48130/VR-2023-0031.

[CR39] Yoo SD, Cho YH, Sheen J. Arabidopsis mesophyll protoplasts: a versatile cell system for transient gene expression analysis. Nat Protoc. 2007;2:1565–72. 17585298 10.1038/nprot.2007.199

[CR40] Zandalinas SI, Fritschi FB, Mittler R. Global Warming, Climate Change, and Environmental Pollution: Recipe for a Multifactorial Stress Combination Disaster. Trends Plant Sci. 2021;26:588–99. 33745784 10.1016/j.tplants.2021.02.011

[CR41] Zhang YY, Xie YF, Shi HF, Zhuang YF, Zheng Y, Lin HH and Zhou HP. MYB30 Regulates Submergence Tolerance by Repressing Ethylene Biosynthesis via ACS7 in Arabidopsis. Plant Cell Physiol. 2023;64(7):814–25. 10.1093/pcp/pcad041. 10.1093/pcp/pcad041 37148388

[CR42] Zhao N, Li C, Yan Y, Cao W, Song A, Wang H, Chen S, Jiang J, Chen F. Comparative transcriptome analysis of waterlogging-sensitive and waterlogging-tolerant chrysanthemum morifolium cultivars under waterlogging stress and reoxygenation conditions. Int J Mol Sci. 2018;19:1455. 29757964 10.3390/ijms19051455 PMC5983694

[CR43] Zhou Y, Xiong Q, Yin C, Ma B, Chen S and Zhang J. Ethylene biosynthesis, signaling, and crosstalk with other hormones in rice. Small Methods. 2020;4:1900278. 10.1002/smtd.201900278.

[CR44] Zhu S, Liu M, Zhou J. Inhibition by nitric oxide of ethylene biosynthesis and lipoxygenase activity in peach fruit during storage. Postharvest Biol Tec. 2006;42:41–8.

