# Supplementary material for: CmHRE2L-CmACS6 transcriptional cascade negatively regulates waterlogging tolerance in Chrysanthemum
Source: Mol Hortic. 2025 Mar 3;5:15. doi: 10.1186/s43897-024-00138-8 (PMC11874658; doi:10.1186/s43897-024-00138-8)
Supplement: Supplementary file 1 — Supplementary Material 1: Figure S1 Heatmap analysis of DEGs in 'Qinglu' and 'Nannong Xuefeng' related to ACS genes (Zhao et al.,2018). Figure S2 Cloning of CmACS6 and characterization of CmACS6 protein in chrysanthemum. Figure S3 Identification of CmACS6 transgenic lines. Figure S4 The performance of CmACS6 transgenic lines under inundation conditions. Figure S5 The root system of CmACS6 transgenic lines after waterlogging. Figure S6 SDS-PAGE analysis of DNA affinity trapping. [file 43897_2024_138_MOESM1_ESM.docx]

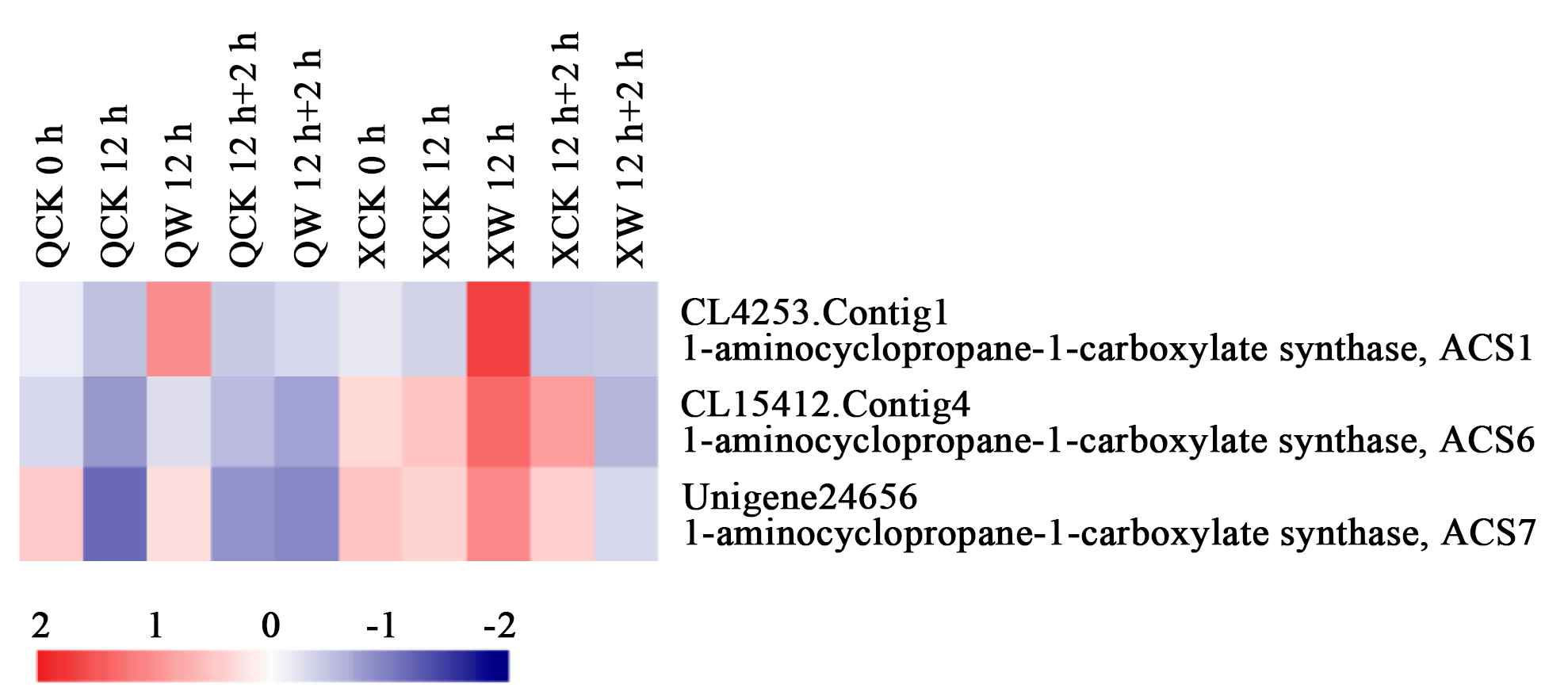


Figure S1 Heatmap analysis of DEGs in 'Qinglu' and 'Nannong Xuefeng' related to ACS genes (Zhao *et al.*, 2018). The bar represents the scale of the expression levels of each gene (log_2_FPKM, fragments per kilobase of transcript per million fragments mapped) in each sample, as indicated by red/blue rectangles. Red rectangles represent the high of genes expression, and blue rectangles represent low level of gene expression. Q: ‘Qinglu’; X: ‘Nannong Xuefeng’. CK: control; WL: waterlogging. W 12 h: waterlogging treat for 12 h; W 12 h+2 h: waterlogging 12 h followed by 2 h reoxygenation recovery.


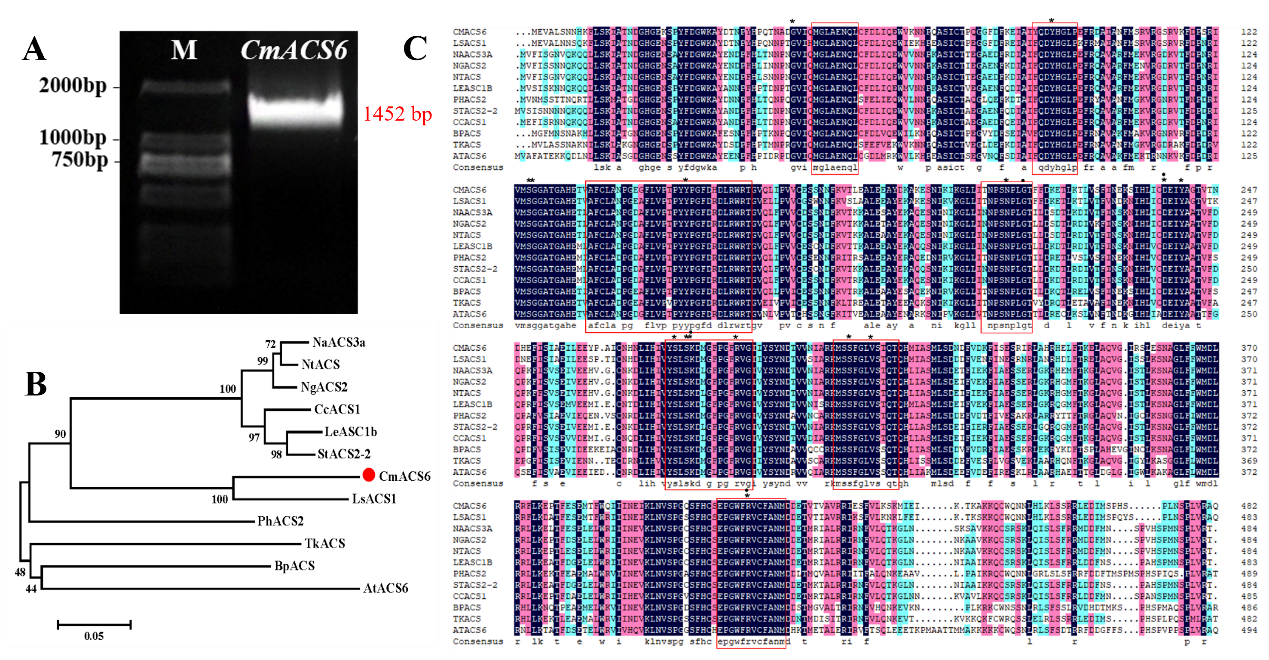


Figure S2 Cloning of *CmACS6* and characterization of CmACS6 protein in chrysanthemum. A, DNA gel electrophoresis results of *CmACS6* gene cloning. M: DL2000. B, Phylogenetic tree of CmACS6 and its homologs from various plant species. Note: LsACS1 (*Lactuca sativa*, AAP14019); NaACS3a (*Nicotiana attenuate*, AAR99392); NgACS2 (*Nicotiana glutinosa*, AAC83147); NtACS (*Nicotiana tabacum*, CAA06288); LeASC1b (*Lycopersicon esculentum*, AAB17279); PhACS2 (*Pelargonium hortorum*, AAB70885); StACS2-2 (*Solanum tuberosum*, WDV57381); Capsicum chinense CcACS1 (BAG30909); BpACS (*Betula platyphylla*, UVJ47666); TkACS (*Trichosanthes kirilowii*, ALR99803); AtACS6 (*Arabidopsis thaliana*, GAY39182). C, Multiple sequence alignment analysis of the amino acid sequences of CmACS6, LsACS1, NaACS3a, NgACS2, NtACS, LeACS1b, PhACS2, StACS2-2, CcACS1, BpACS, TkACS and AtACS6.


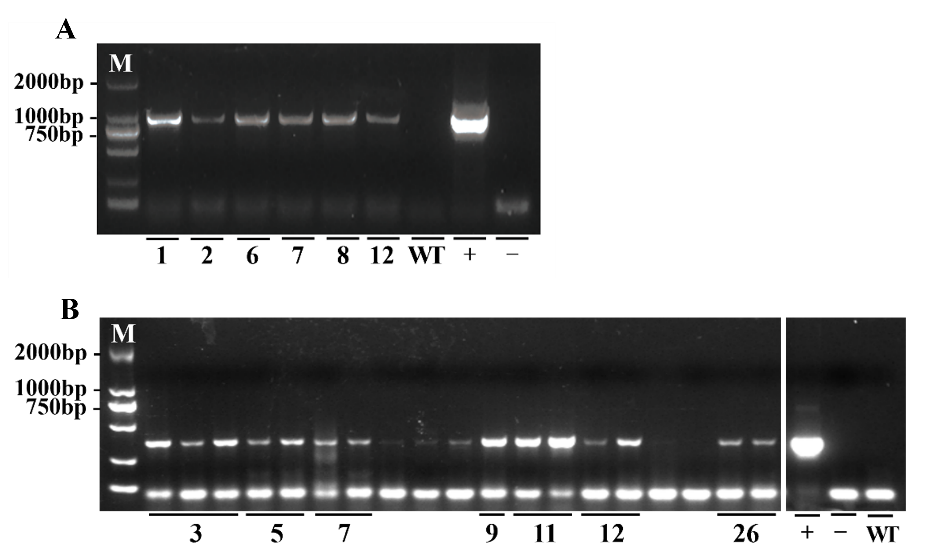


Figure S3 Identification of *CmACS6* transgenic lines. A, *CmACS6-like* overexpression lines. B, *CmACS6-like* silensing lines. M: DL2000. Numbers represent different transgenic lines. +: Positive control; -: Negative control; WT: Wild-type ‘Jinba’.


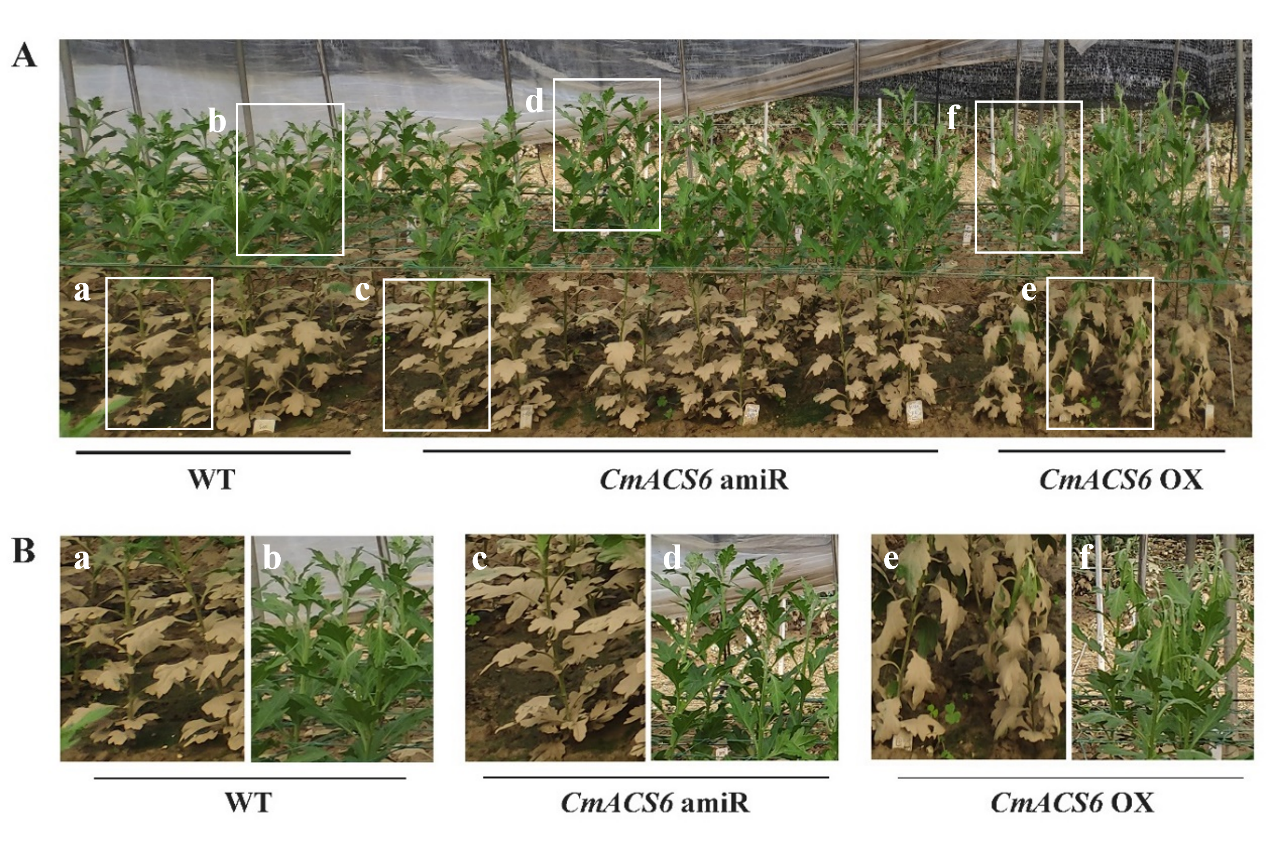


Figure S4 The performance of *CmACS6* transgenic lines under inundation conditions. A, Phenotypes of *CmACS6* transgenic lines in inundation. Bar: 10 cm. B, Local enlarged graph of figure A. Different lowercase letters indicate the different magnification areas. The yellow coloration of the lower middle portion of the plant leaves is a result of immersion in muddy water during water logging and is not due to waterlogging stress injury. WT: wild type; *CmACS6* amiR: *CmACS6* amiRNA lines; *CmACS6* OX: *CmACS6* overexpression lines.


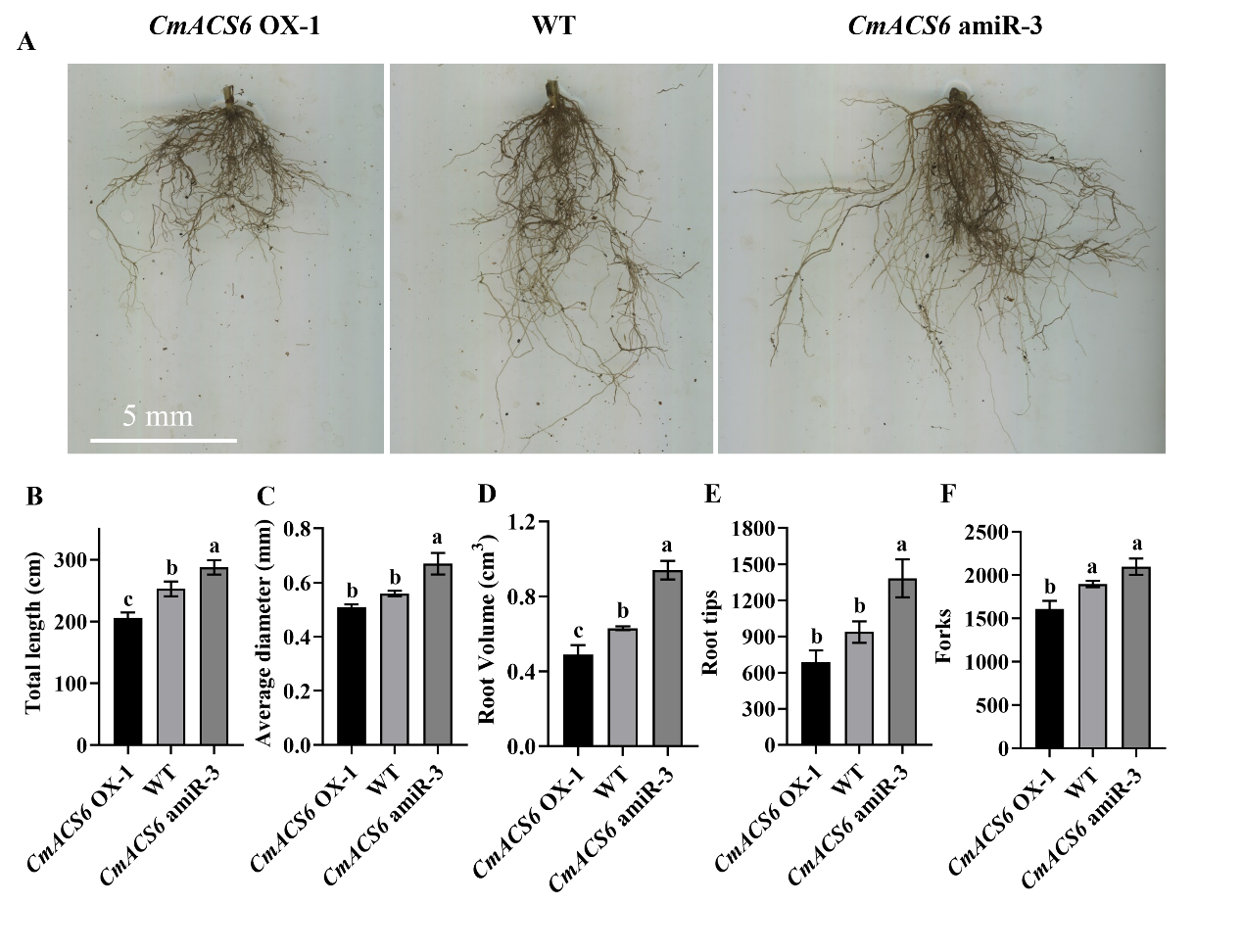


Figure S5 The root system of CmACS6 transgenic lines after waterlogging. A, Phenotypes of roots after waterlogging. Bar: 5 cm. B-F, Data analysis of root conditions after waterlogging. B, Total length of roots. C. Average diameter of roots. D. Root Volume. E, Numbers of root tips F, Numbers of root forks. WT: wild type; *CmACS6* amiR: *CmACS6* amiRNA lines; *CmACS6* OX: *CmACS6* overexpression lines. Error bars refer to standard deviation (SD, n = 3). Different lowercase letters indicate significant differences at P < 0.05 (ANOVA, Turkey’s correction).


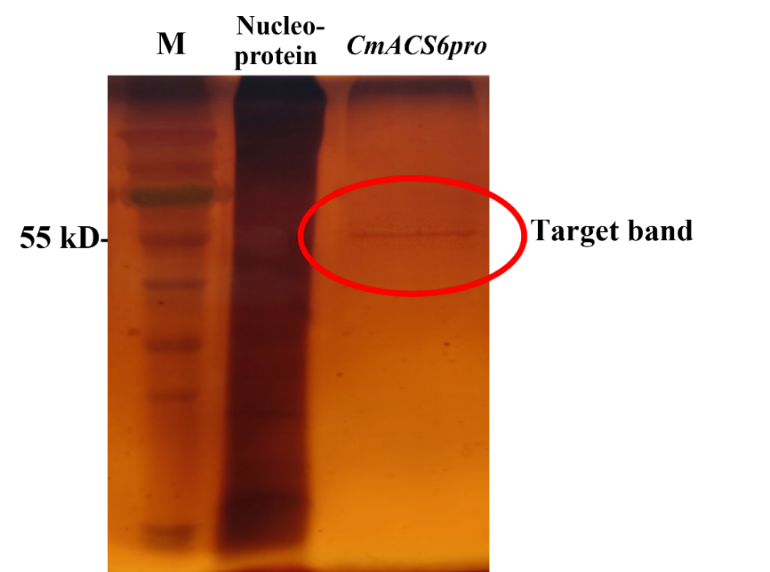


Figure S6 SDS-PAGE analysis of DNA affinity trapping. M: Marker；Nucleoprotein: Nucleoprotein；CmACS6pro: Cis-element 3 in *CmACS6pro*.
